# Supplementary material for: Origins of chromosome instability unveiled by coupled imaging and genomics
Source: Nature. 2025 Oct 29;648(8093):383–93. doi: 10.1038/s41586-025-09632-5 (PMC12695650; doi:10.1038/s41586-025-09632-5)
Supplement: Supplementary file 1 — This file contains Supplementary Figs. 1–11, descriptions for Tables 1–17 (supplied as a separate spreadsheet), Methods and Notes. [file 41586_2025_9632_MOESM1_ESM.pdf]

nature portfolio

https://doi.org/10.1038/s41586-025-09632-5

Supplementary information

Origins of chromosome instability unveiled by coupled imaging and genomics

In the format provided by the authors and unedited

SUPPLEMENTARY INFORMATION

Origins of chromosome instability unveiled by coupled imaging and genomics

Marco Raffaele Cosenza[1], Alice Gaiatto[1], Büşra Erarslan Uysal[1,2,3], Álvaro Andrades[1], Nina Luisa Sautter[1], Marina Simunovic[1], Michael Adrian Jendrusch[1], Sonia Zumalave[4], Tobias Rausch[1,5], Aliaksandr Halavatyi[6], Eva-Maria Geissen[7], Joshua Lucas Eigenmann[1], Thomas Weber[1,7], Patrick Hasenfeld[1], Eva Benito[1], Catherine Stober[1], Isidro Cortes-Ciriano[4], Andreas E. Kulozik[2,3], Rainer Pepperkok[6,8], Jan O. Korbel[1,2,4,9,@]

[1]Genome Biology Unit, European Molecular Biology Laboratory (EMBL), Heidelberg, Germany.
[2]Molecular Medicine Partnership Unit (MMPU), EMBL, University of Heidelberg, Heidelberg, Germany.
[3]Department of Pediatric Oncology, Hematology, and Immunology, University of Heidelberg and Hopp Children's Cancer Center, Heidelberg, Germany and CCU Pediatric Leukemia, German Cancer Research Center (DKFZ), Heidelberg, Germany.
[4]European Bioinformatics Institute (EMBL-EBI), Hinxton, Cambridgeshire, UK.
[5]Genomics Core Facility, EMBL, Heidelberg, Germany.
[6]Advanced Light Microscopy Core Facility, EMBL, Heidelberg, Germany.
[7]Data Science Centre, EMBL, Heidelberg, Germany.
[8]Cell Biology and Biophysics Unit, EMBL, Heidelberg, Germany.
[9]Bridging Research Division on Mechanisms of Genomic Variation and Data Science, German Cancer Research Center (DKFZ), Heidelberg, Germany.

[@]Correspondence should be addressed to Jan.Korbel@embl.de

SUPPLEMENTARY INFORMATION 1
Supplementary Figures 3
Supplementary Tables 17
Supplementary Methods 18
Photolabeling strategies 18
Microscope automation and imaging 18
Online image analysis with magic_tools 19
XGBoost classifier training 20
Convolutional neural network based micronucleus classifier training 21
Strandtools – an optimised single-cell CA calling algorithm for Strand-seq data 21
Code reproducibility 22
Computational sister cell pair discovery from Strand-seq data 22
Designing single-guide RNA 22
Statistical testing for biases in CA frequencies, SCEs and breakpoint locations 23
CA rate estimation by bound-constrained minimization 24
Western blotting 24
Supplementary Notes 25
Limitations in detection of micronuclei 25
Differential expression analysis of micronucleated cells with MAGIC 26
Pan-cancer WGS cohorts 26
Comparing copy-number features from spontaneous micronuclei with the PCAWG resource 27
Quantification of aneuploidies in the PCAWG resource 29
Inference of isochromosomes using bulk WGS data 30
References 32

Supplementary Figures

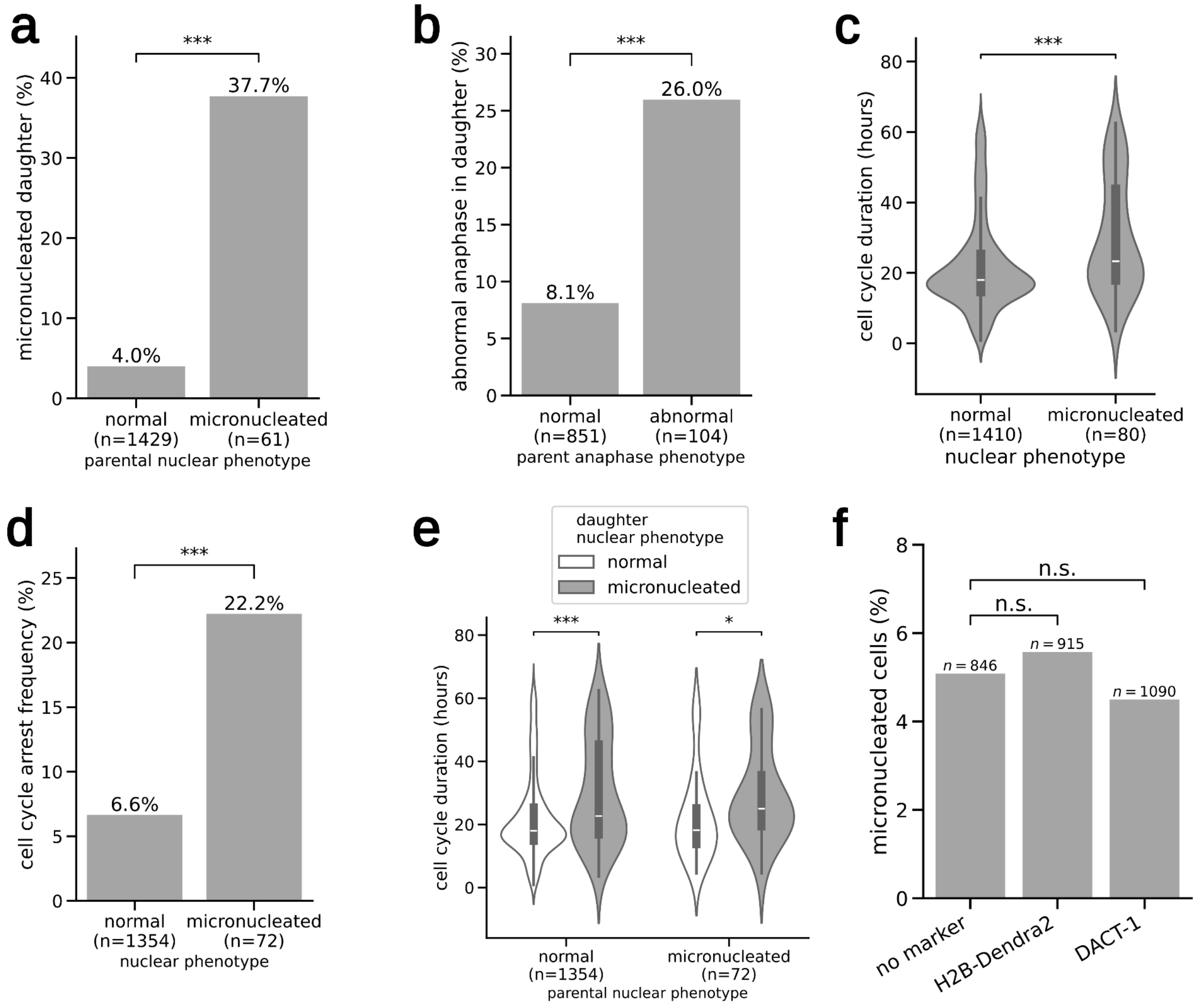

Supplementary Figure 1 | Long-term live-cell imaging analysis in MCF10A cells

(a) Frequency of micronucleated parent cells that generate at least one micronucleated daughter (Fisher's exact test, $P < 0.001$). (b) Frequency of abnormal anaphase in daughter cells, depending on the preceding anaphase phenotype of the parent cell (Fisher's exact test, $P < 0.001$). (c) Cell cycle duration in hours for daughter cell generation. Duration measured from mitosis to mitosis (Mann-Whitney U test, two-sided, $P < 0.001$). (d) Frequency of cell cycle arrest, defined as cells with a cell cycle duration longer than the 99th percentile (Fisher's exact test $P < 0.001$). (e) Cell cycle duration of daughter cells depending on the nuclear phenotype seen in the parental generation (Mann-Whitney U test, two-sided, normal $P < 0.001$, micronucleated $P < 0.05$). (f) Frequency of micronucleated cells in the parental MCF10A cell line (no marker), in the H2B-Dendra2 expressing clone and following DACT-1 exposure (Fisher's exact test, n.s.: not significant). For significance levels see ***Methods****, * P<0.05, **P<0.01, ***P<0.001.*

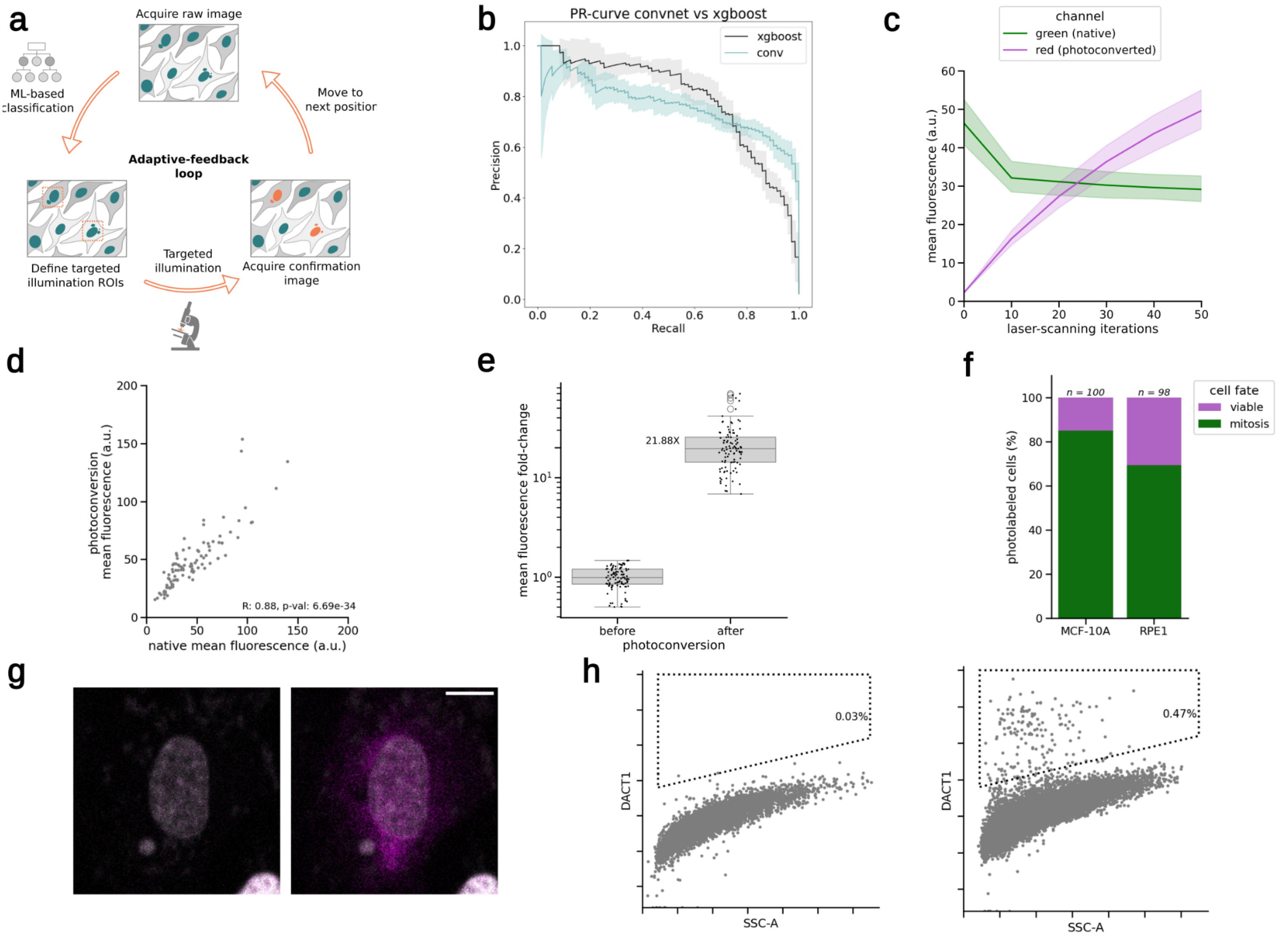

Supplementary Figure 2 | Establishing imaging parameters for setting up MAGIC

(a) Schematics depicting the adaptive-feedback loop at the core of MAGIC's microscope automation. (b) Precision-recall curves for XGBoost (grey) and convolutional (conv, cyan) neural network models for micronuclei classification. Coloured bar represents standard deviation of performance on five different training-test dataset splits. (c) Photoconversion dynamics of targeted illumination of H2B-Dendra2. Mean fluorescence was measured in 151 cells every 10 laser-scanning iterations. Color band, 95% confidence interval. (d) Correlation between native (green) and photoconversion (red) mean fluorescence of photolabeled nuclei in MCF10A cells expressing H2B-Dendra2 (Pearson correlation coefficient) (e) Fold change in mean nucleus fluorescence before and after photolabeling in MCF10A cells expressing H2B-Dendra2. Center line, median; box limits, upper and lower quartiles; whiskers, 1.5x interquartile range; points, outliers. (f) Fate of imaged cells followed for 24 hours after photolabeling (g) RPE-1 cells before (left) and (after) photoactivation DACT-1 dye (magenta). Nuclear DNA (grey) visualised by NucSpot 650. Scale bar: 10 µm (h) FACS profile of RPE-1 cells in control (left) and photolabeled (right) conditions. Side scatter signal area (SSC-A) versus DACT-1 fluorescence is plotted, and the sorting gate is represented by a dashed line.

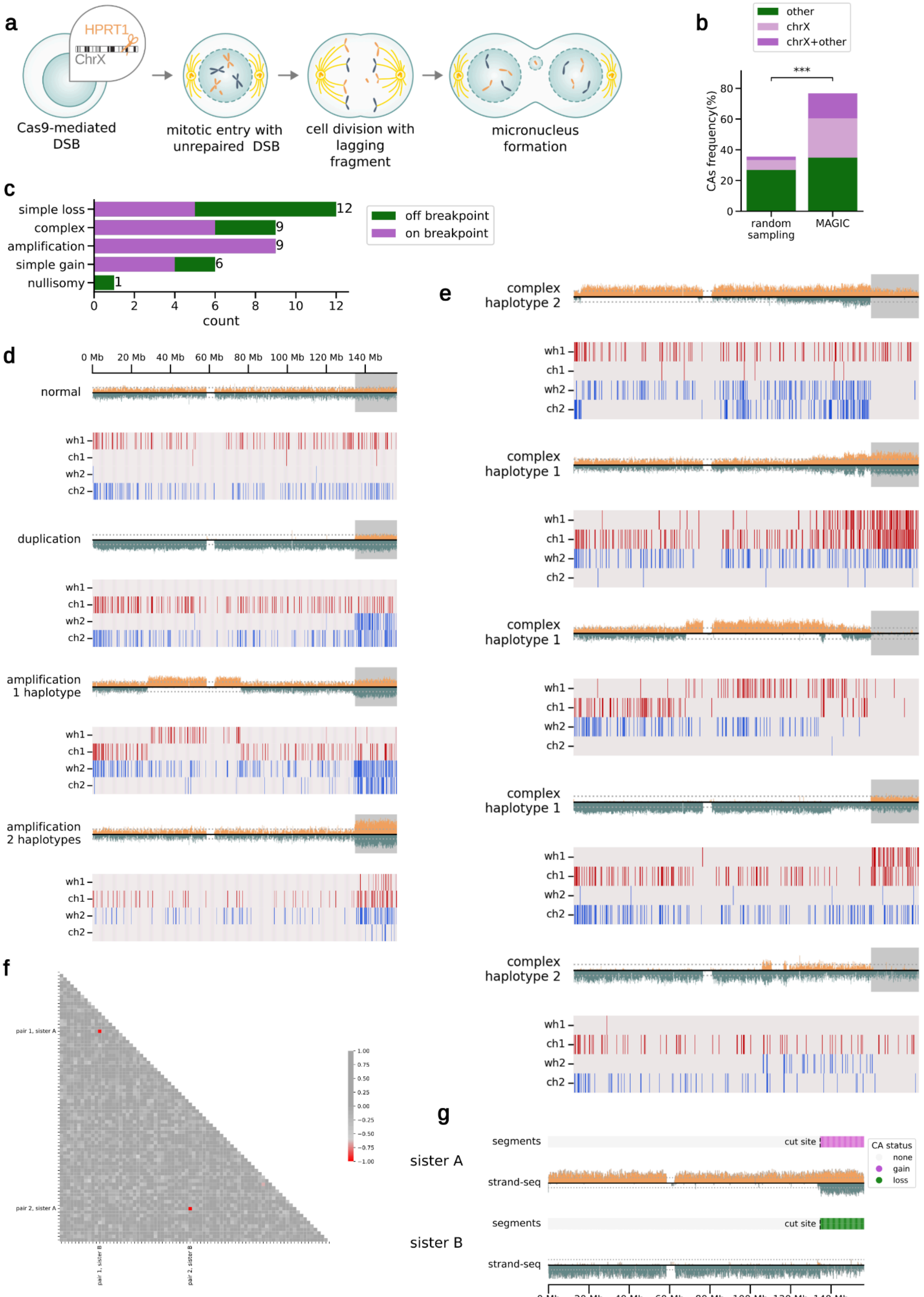
a
HPRT1
ChrX
Cas9-mediated DSB
mitotic entry with unrepaired DSB
cell division with lagging fragment
micronucleus formation
b
other
chrX
chrX+other
***
CAs frequency(%)
0
20
40
60
80
random sampling
MAGIC
c
simple loss
complex
amplification
simple gain
nullisomy
12
9
9
6
1
0
2
4
6
8
10
12
count
off breakpoint
on breakpoint
d
0 Mb
20 Mb
40 Mb
60 Mb
80 Mb
100 Mb
120 Mb
140 Mb
normal
wh1
ch1
wh2
ch2
duplication
amplification 1 haplotype
amplification 2 haplotypes
e
complex haplotype 2
complex haplotype 1
f
pair 1, sister A
pair 2, sister A
pair 1, sister B
pair 2, sister B
1.00
0.75
0.50
0.25
0.00
−0.25
−0.50
−0.75
−1.00
g
sister A
sister B
segments
strand-seq
cut site
CA status
none
gain
loss
0 Mb
20 Mb
40 Mb
60 Mb
80 Mb
100 Mb
120 Mb
140 Mb

Supplementary Figure 3 | Verification of the MAGIC platform through Cas9-induced CAs

(a) Schematic showing how Cas9-mediated DSB can lead to micronucleus formation. In this example, Cas9 cuts at the HPRT1 locus on one chromatid of chromosome X in G2 phase, generating an acentric chromosome fragment lacking the centromere. If the cell enters mitosis without repairing the DSB, the acentric fragment cannot participate in mitosis as it would be missing the kinetochore necessary for spindle orientation. As a consequence, the acentric fragment would lag behind at anaphase and be inherited by either daughter cell, forming a micronucleus. (b) Frequency of CAs in single cells randomly sampled, or selected using MAGIC. chrX: chromosome X (Fisher's exact test, $P<0.001$). (c) Breakdown of copy-number alterations after targeted DSB induction at the HPRT1 locus. Simple loss and simple gain indicate an isolated change in copy-number. Amplification refers to all isolated copy-number gains of two or more. Nullisomy is the isolated and complete loss of the chromosome segment. Complex CAs are defined as events that involve more than 2 breakpoints, which can comprise combinations of copy-number gains and/or losses. (d,e) Strand-seq based haplotype-aware analysis[1] of acentric fragment copy-number gains and amplifications of the cut-off segment (d) and complex CA affecting a single haplotype (e). Grey background in strand-seq plots: cut-off fragment. Phased haplotags[1] are represented by red or blue lines for haplotype 1 and 2, respectively. wh1: watson strand, haplotype 1. wh2: watson strand, haplotype 2. ch1: crick strand, haplotype 1. ch2: crick strand, haplotype 2. (f) Strand orientation state correlation between single cells as a predictor for sister cell pair relationship. Two sister cell pairs displaying a pronounced negative correlation are shown in red (Pearson correlation coefficient; scale bar shown to the right). (g) Example of a reciprocal CA in sister cells. Sister A carries a copy-number gain of the cut-off fragment generated by the cut (top), while sister B carries a copy-number loss for the same segment (bottom). Cut site annotated by dashed line.

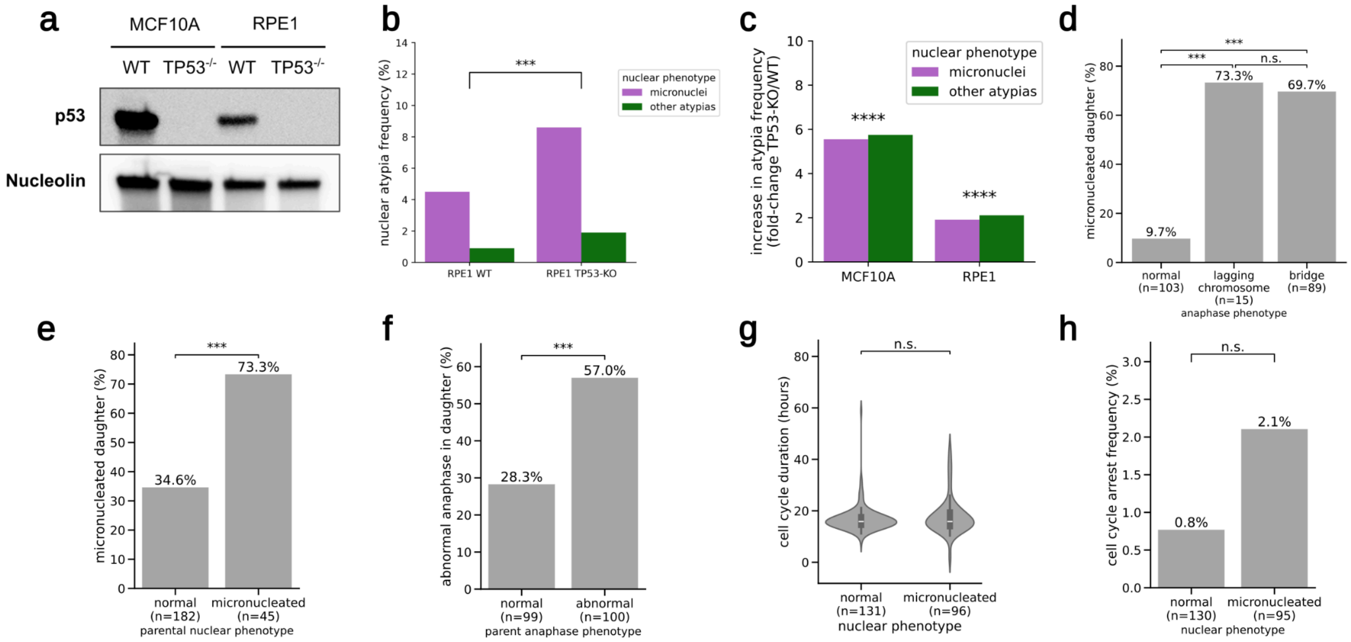

Supplementary Figure 4 | Characterisation of the *TP53-/-* cell line models used in this study

(a) Western blot for the p53 protein, with nucleolin used as loading control. (b) Nuclear atypia frequency in RPE-1 WT and TP53-/- cell line models (Fisher's exact test). (c) Fold-increase in nuclear atypia frequency of TP53-/- models compared to their WT counterparts (Fisher's exact test, Bonferroni corrected, P<0.0001). (d-h) Live-cell imaging analysis of MCF10A TP53-/- cells. (d) Frequency of daughter micronucleation associated with different anaphase phenotypes (Fisher's exact test, n.s.: not significant). (e) Frequency of micronucleated parent cells that generate at least one micronucleated daughter (Fisher's exact test). (f) Frequency of abnormal anaphases in daughter cells depending on the anaphase phenotype of the parent cell (Fisher's exact test). (g) Cell cycle duration in hours for daughter cell generation. Duration measured from mitosis to mitosis (Mann-Whitney U test, two-sided, n.s.: not significant). Center line, median; inner box limits, upper and lower quartiles; whiskers, 1.5x interquartile range; gray patch, kernel density estimate. (h) Frequency of cell cycle arrest, defined as cells with a cell cycle duration longer than the 99th percentile (Fisher's exact test, n.s.: not significant).

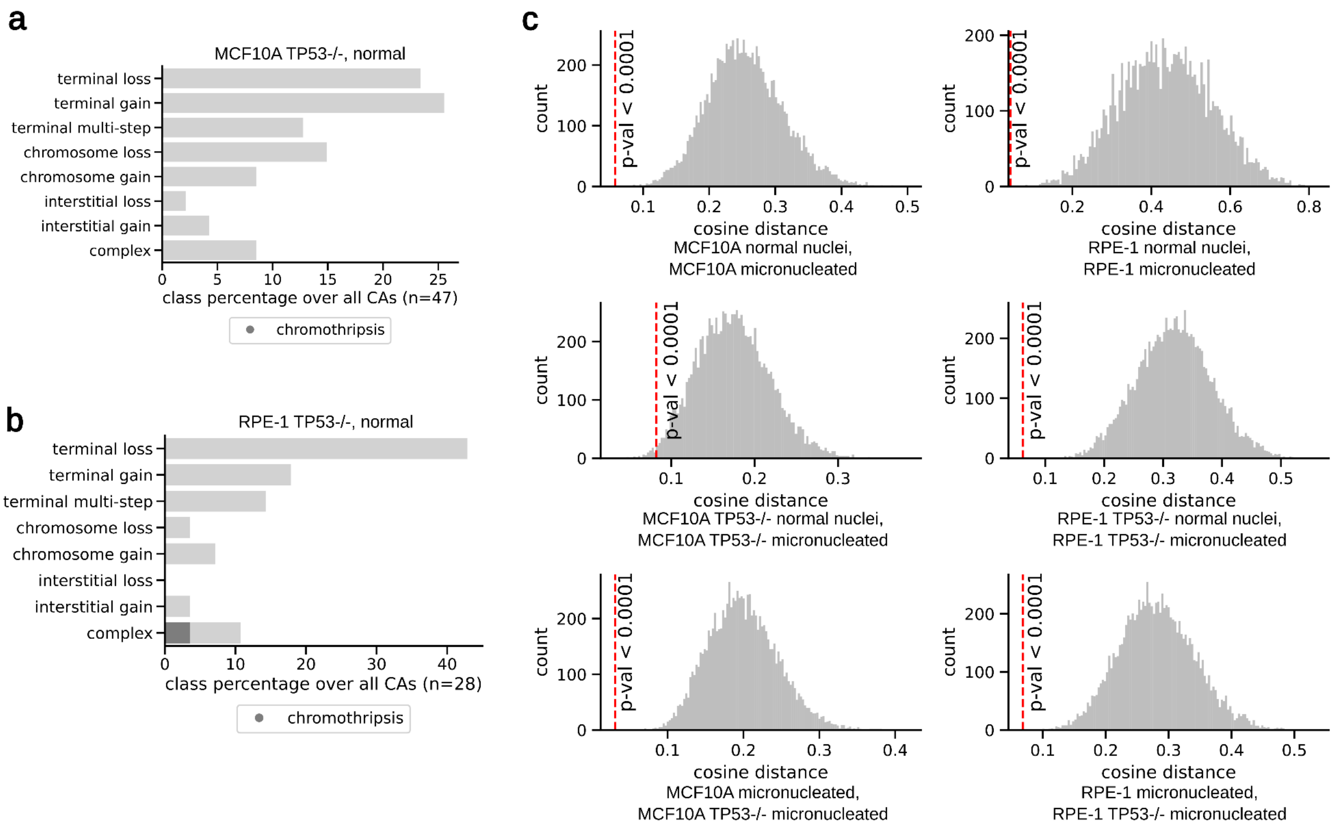

Supplementary Figure 5 | CA landscape characterization in the *TP53-/-* cell line models

Breakdown of CA classes in normal (i.e. not micronucleated) MCF10A (a) and RPE-1 (b) TP53-/- cells, with the class percentage shown in comparison to all CAs detected. (c) Distribution derived from permutation of CA class labels in sample pairs, with the cosine distance statistic (see ***Methods****) used to evaluate CA class distribution similarity. Gray histogram: expected distribution of cosine distance between the reference sample and the permuted one. Red dashed line: observed statistic. The observed cosine distance is significantly smaller than expected under the permutation, implying high similarity of CA class distributions across experimental conditions.*

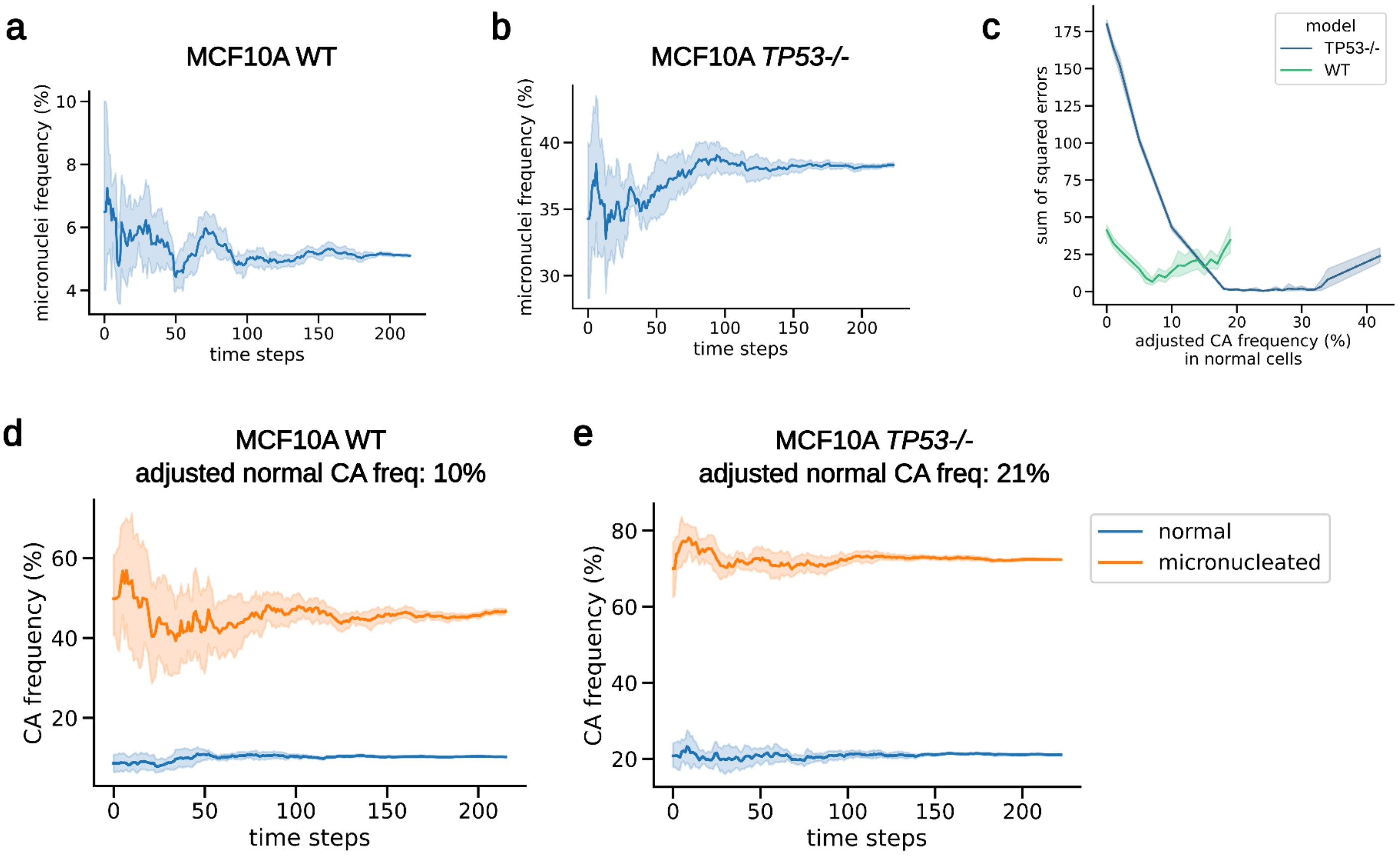

Supplementary Figure 6 | Characterization of the agent-based model for CA rate estimation
Simulated micronuclei frequency for the MCF10A WT (a) and TP53-/- (b) models. Coloured band: 95% confidence interval calculated over 10 simulations. (c) Goodness-of-fit optimization for MCF10A WT and TP53-/- models with different adjusted levels of CA frequency. For each frequency level tested, optimisation was run starting from 50 initialisations using uniform random numbers. Simulated CA frequency for MCF10A WT (d) and TP53-/- (e) models. Coloured bands: 95% confidence interval calculated over 10 simulations.

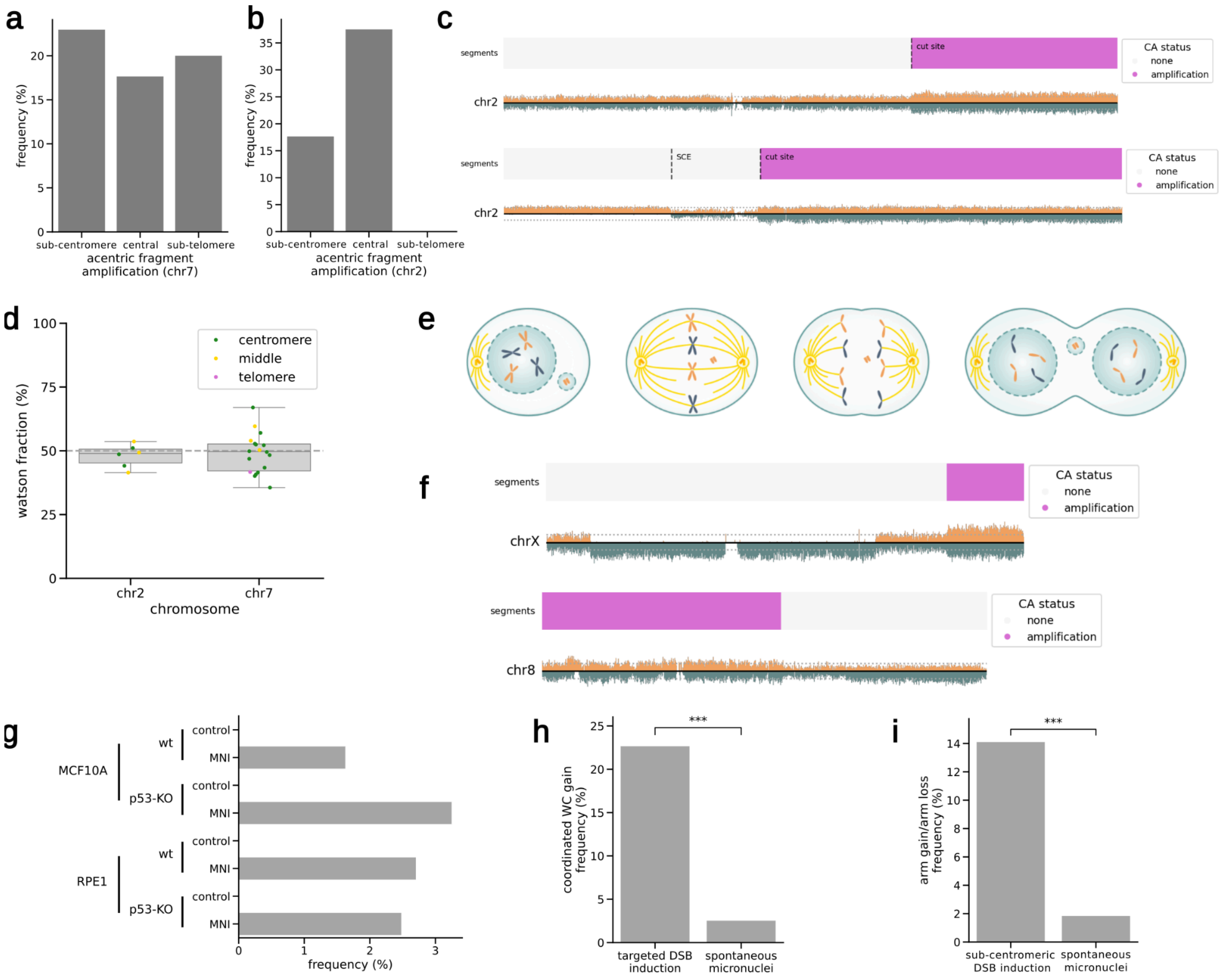

Supplementary Figure 7 | Analysis of acentric fragment amplifications

Frequency of observed acentric fragment amplifications in our targeted DSB experiments for chromosome 7 (a) and chromosome 2 (b). Examples of acentric fragment amplification in spontaneously formed micronuclei (f) and following targeted DSB induction (c). (d) Watson read fraction of amplified acentric fragments. Center line, median; box limits, upper and lower quartiles; whiskers, 1.5x interquartile range; points, outliers. (e) Scheme showing how acentric fragments contained in micronuclei do not participate in mitosis and can be inherited as a single unit. (g) frequency of acentric amplification in spontaneous micronuclei across cell lines and nuclear phenotypes. MNI: micronuclei. Frequency of acentric amplification (h) and arm gain/arm loss (i) in spontaneous micronuclei when compared to targeted DSB induction (significance testing based on Fisher's exact test).

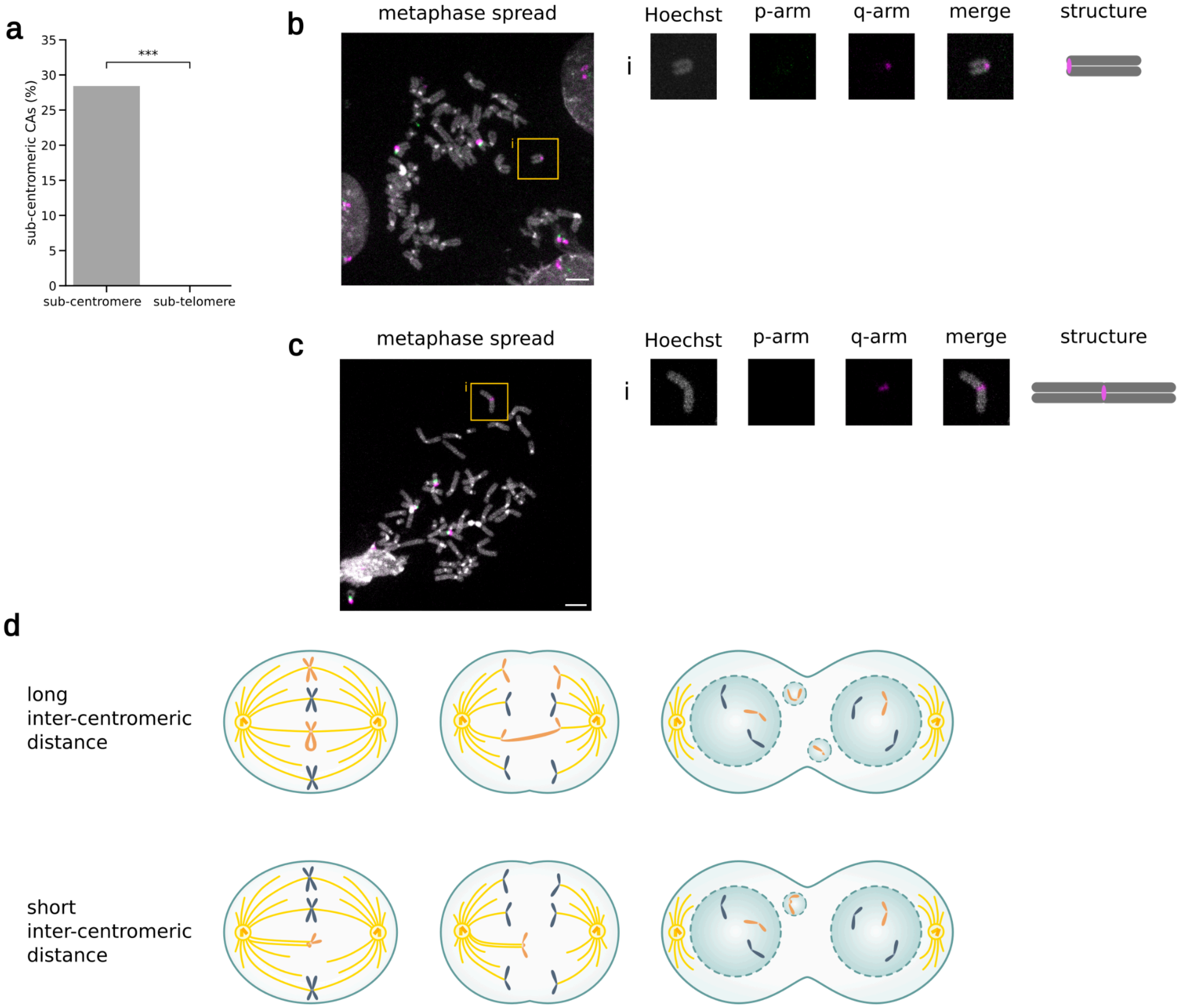

Supplementary Figure 8 | Acentric chromosome derivative and mechanism of isochromosome generation

(a) Frequency of abnormal sub-centromeric probe hybridisation patterns across different cut-sites (Fisher's exact test). (b) Metaphase spread example with abnormal chromosome 7 structures. Right: magnified regions of interest (ROIs), with breakdown per fluorescent channel and putative chromosome structures. ROIs descriptions - i: cut fragment constituting the q-arms of chromosome 7 and a probe signal at one extremity. (c) Metaphase spread example with abnormal chromosome 7 structures. ROI i: isoacentric chromosome, with similar sized segments around the middle q-arm signal. Scale bars 5µm. (d) Mitosis schemes with isodicentric chromosomes, contrasting mitosis with chromatin bridge formation from long-intercentromeric distance (top) and isochromosome formation with short-intercentromeric distance (bottom). We note that isodicentric chromosomes might still form micronuclei following their formation, due to potential occasional erroneous kinetochore-microtubule attachments. One possibility is that isodicentrics might form monotelic spindle attachments, leading to misaligned chromosomes that satisfy the spindle assembly checkpoint and micronucleation[2].

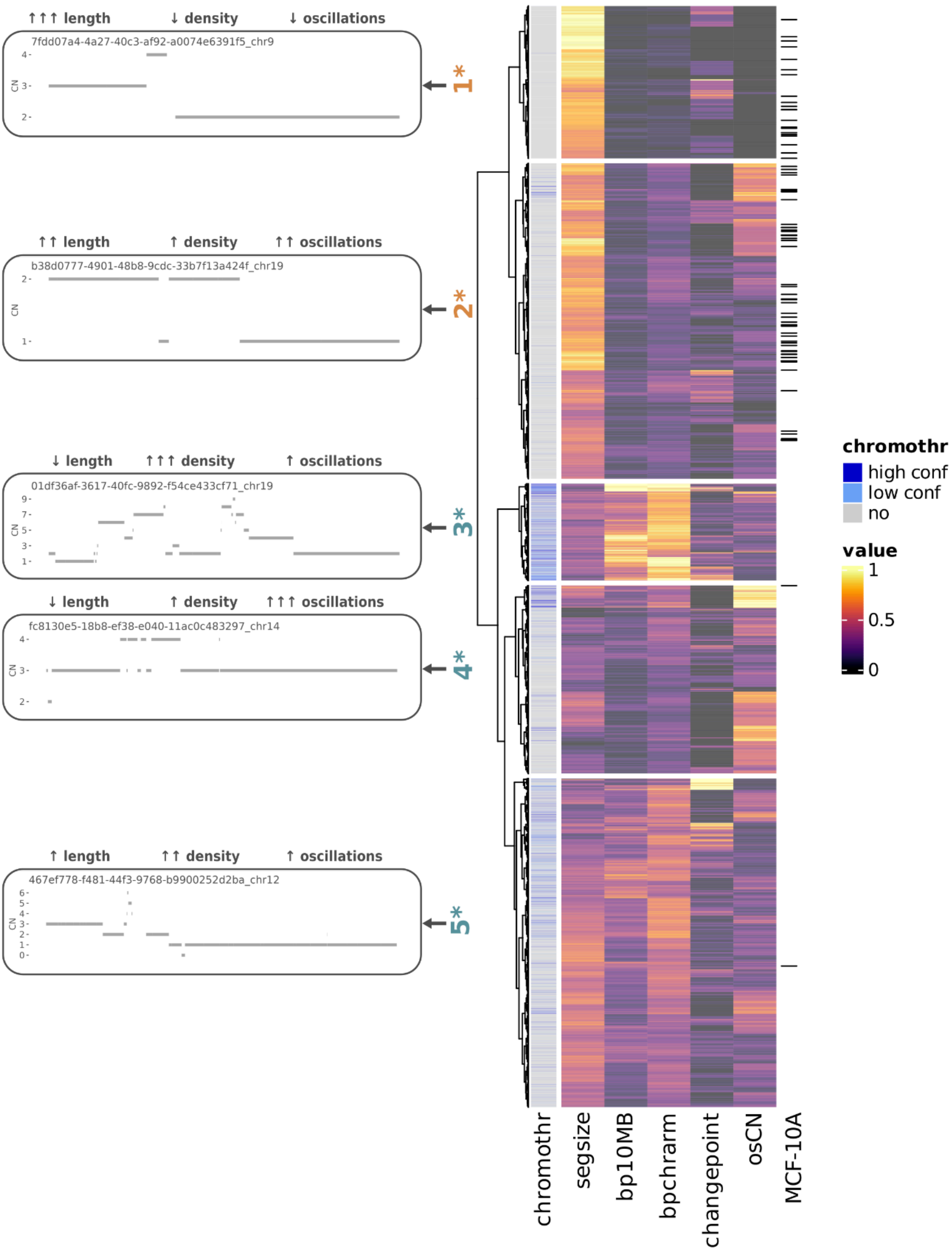
↑↑↑ length ↓ density ↓ oscillations
7fdd07a4-4a27-40c3-af92-a0074e6391f5_chr9
1*
↑↑ length ↑ density ↑↑ oscillations
b38d0777-4901-48b8-9cdc-33b7f13a424f_chr19
2*
↓ length ↑↑↑ density ↑ oscillations
01df36af-3617-40fc-9892-f54ce433cf71_chr19
3*
↓ length ↑ density ↑↑↑ oscillations
fc8130e5-18b8-ef38-e040-11ac0c483297_chr14
4*
↑ length ↑↑ density ↑ oscillations
467ef778-f481-44f3-9768-b9900252d2ba_chr12
5*
CN
chromothr
high conf
low conf
no
value
1
0.5
0
chromothr
segsize
bp10MB
bpchrarm
changepoint
osCN
MCF-10A

Supplementary Figure 9 | Copy-number alterations clustering based on copy-number (CN) features

Heatmap of CN features across PCAWG and MCF10A chromosomes with at least 2 non-diploid segments. MCF10A chromosomes are highlighted with ticks on the right side. Chromosomes are clustered by Euclidean distances and ward.D2 linkage. For visualisation purposes, 99% winsorization was applied prior to normalisation. Inferred chromothripsis events are shown on the left column bar. For PCAWG, high as well as low confidence chromothripsis calls were considered[3]. For MCF10A, we considered all of our manually curated calls (we note that owing to the single cell nature of the MCF10A data with much fewer reads generated per genome, short chromothripsis-derived DNA fragments <200kb in size are likely to remain undetected by Strand-seq). For each cluster number, asterisks denote significant enrichment (orange) or depletion (turquoise) of MCF10A chromosomes in each cluster, as assessed by a permutation test (FDR-adjusted $p < 0.05$). To the left of each cluster, a schematic of the CN patterns of a representative chromosome is shown. Chromothr, chromothripsis; segsize, length of non-diploid segments; bp10MB, number of breakpoints per 10 Mb; bpchrarm, number of breakpoints per chromosome arm; changepoint, magnitude of the changepoints, osCN, number of CN oscillations.

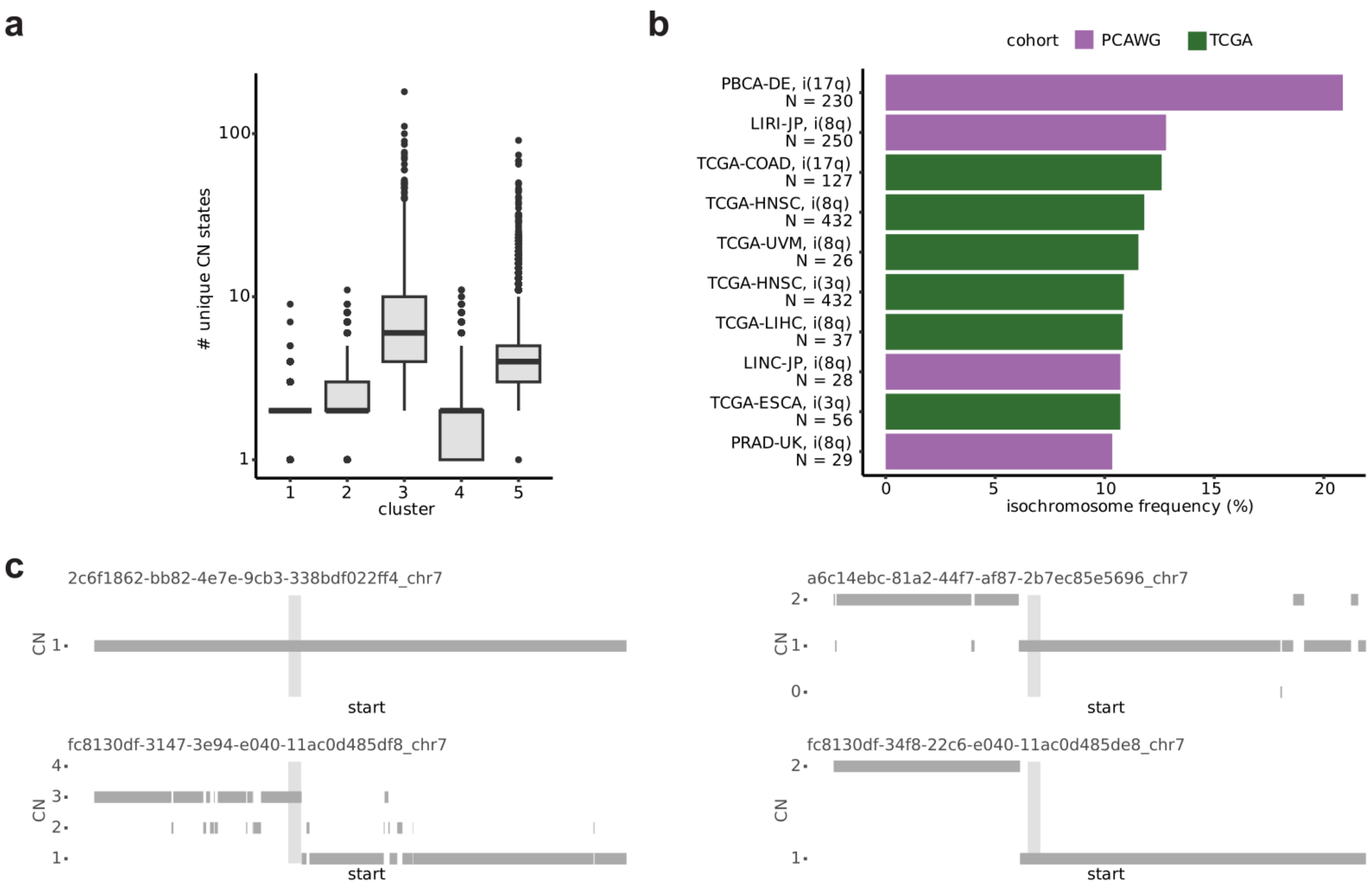

Supplementary Figure 10 | Ancillary copy-number and isochromosome analyses in primary cancer genome datasets

(a) Number of unique CN states per chromosome in the PCAWG dataset. Chromosomes are grouped by clusters as defined in ***Supplementary Figure 9****. Center line, median; box limits, upper and lower quartiles; whiskers, 1.5x interquartile range; points, outliers. (b) The top 10 most frequent isochromosomes identified in TCGA and PCAWG sub-cohorts with more than 25 donors. PBCA: paediatric brain cancer; LIRI: hepatocellular carcinoma; COAD: colon adenocarcinoma; HNSC: head and neck squamous cell carcinoma; UVM: uveal melanoma; LIHC / LINC: liver hepatocellular carcinoma; ESCA: Esophageal Carcinoma; PRAD: prostate adenocarcinoma. (c) Examples of losses affecting the q-arm of chromosome 7 in breast cancer samples from the PCAWG cohort.*

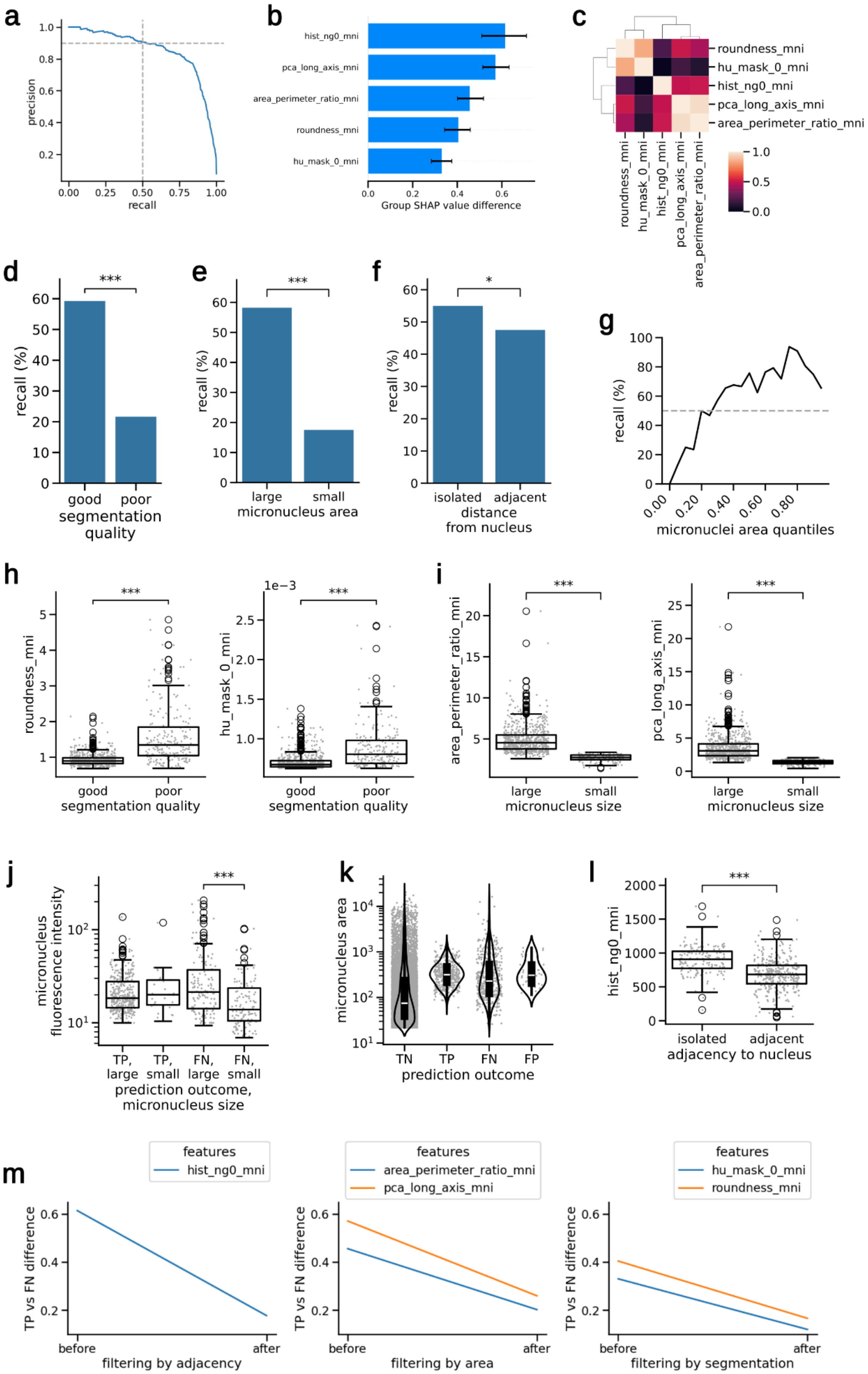

a
precision
recall
b
hist_ng0_mni
pca_long_axis_mni
area_perimeter_ratio_mni
roundness_mni
hu_mask_0_mni
Group SHAP value difference
c
roundness_mni
hu_mask_0_mni
hist_ng0_mni
pca_long_axis_mni
area_perimeter_ratio_mni
d
recall (%)
***
good
poor
segmentation quality
e
***
large
small
micronucleus area
f
*
isolated
adjacent
distance from nucleus
g
recall (%)
micronuclei area quantiles
h
roundness_mni
hu_mask_0_mni
good
poor
segmentation quality
i
area_perimeter_ratio_mni
pca_long_axis_mni
large
small
micronucleus size
j
micronucleus fluorescence intensity
TP, large
TP, small
FN, large
FN, small
prediction outcome, micronucleus size
k
micronucleus area
TN
TP
FN
FP
prediction outcome
l
hist_ng0_mni
isolated
adjacent
adjacency to nucleus
m
features
hist_ng0_mni
area_perimeter_ratio_mni
pca_long_axis_mni
hu_mask_0_mni
roundness_mni
TP vs FN difference
before
after
filtering by adjacency
filtering by area
filtering by segmentation

Supplementary Figure 11 | Exploring limitations in micronuclei detection.
(a) Precision-recall curve calculated on image data from an experiment with MCF10A wild-type, micronucleated cells. (b) Difference in the average SHAP values between false negatives and true positives groups, the bar plot shows the top 5 biased features. (c) Cross-correlation heatmap for the top 5 biased features (Spearman correlation). Difference in recall across the segmentation quality (d), micronucleus area (e) and nucleus adjacency (f) factors (Fisher's exact test). (g) Recall per micronucleus area quantile bins. Value distribution for features related to segmentation quality (h), micronucleus size (i) and adjacency (l). (j) Mean fluorescence intensity of small micronuclei per prediction outcome. (k) Area distribution across prediction outcomes (features compared by Mann Whitney U test). (m) Difference in average SHAP values between false negatives and true positives groups, before and after filtering out adjacent (left panel), small (central panel) or poorly segmented (right panel) micronuclei.

Supplementary Tables

Supplementary Table 1. Summary of single-cell clones analysis by low-pass WGS, with CA annotations. (Table accompanying the manuscript as spreadsheet)

Supplementary Table 2. Summary of sgRNA sequences and PCR primers for the TIDE assay. (Table accompanying the manuscript as spreadsheet)

Supplementary Table 3. Convolutional neural network architecture (Table accompanying the manuscript as spreadsheet)

Supplementary Table 4. Data sources for PCAWG and TCGA analyses. (Table accompanying the manuscript as spreadsheet)

Supplementary Table 5. PCAWG and TCGA cohort identifiers data. (Table accompanying the manuscript as spreadsheet)

Supplementary Table 6. Isochromosome annotation and results. (Table accompanying the manuscript as spreadsheet)

Supplementary Table 7. CA annotations for the targeted DSB-induction MAGIC experiments at the *HPRT1* locus on chromosome X. (Table accompanying the manuscript as spreadsheet)

Supplementary Table 8. CA annotations for the MAGIC experiments performed with the following models: MCF10A and RPE-1 cell lines in their WT and *TP53-/-* models, BJ-5ta and IMR-90 cell lines and MCF10A and RPE-1 exposed to reversine. (Table accompanying the manuscript as spreadsheet)

Supplementary Table 9. CA annotations for targeted DSB-induction MAGIC experiments along chromosome arms 7q and 2q. (Table accompanying the manuscript as spreadsheet)

Supplementary Table 10. Chromosome loss bias analysis by permutation. (Table accompanying the manuscript as spreadsheet)

Supplementary Table 11. Sample metadata for transcriptomic experiment in RPE-1 cells. (Table accompanying the manuscript as spreadsheet)

Supplementary Table 12. Sample metadata for transcriptomic experiment in MCF10A cells. (Table accompanying the manuscript as spreadsheet)

Supplementary Table 13. gene count matrix for transcriptomic experiment in RPE-1 cells. (Table accompanying the manuscript as spreadsheet)

Supplementary Table 14. gene count matrix for transcriptomic experiment in MCF10A cells. (Table accompanying the manuscript as spreadsheet)

Supplementary Table 15. Table of the Top Differentially Expressed Tags in RPE-1 micronucleated cells. (Table accompanying the manuscript as spreadsheet)

Supplementary Table 16. Table of the Top Differentially Expressed Tags in MCF10A micronucleated cells. (Table accompanying the manuscript as spreadsheet)

Supplementary Table 17. Table with links to datasets and repositories associated with this manuscript. (Table accompanying the manuscript as spreadsheet)

Supplementary Methods

Photolabeling strategies

In this study, we employed two photolabeling strategies based on Dendra2 and DACT-1. Dendra2 is a monomeric fluorescent protein, originally derived from *Dendronephthya sp.*, that is amenable to photoconversion with purple-blue light[4]. The photoconversion reaction is irreversible and thus duration of the labeling depends on protein turnover. In our case, we chose to employ H2B-Dendra2, where the photolabeling protein is fused to the histone 2B protein[5]. This enables the visualisation of nuclear atypia, as well as photolabeling. Moreover, thanks to the low turn-over of histone proteins, photolabeling persists also across subsequent generations as demonstrated by the collection and single-cell analysis of sister cells. DACT-1 is a dual-activatable cell tracking molecule previously developed for the controlled and prolonged tagging of single cells[6]. DACT-1 is cell permeable and accumulates inside the cytoplasm thanks to the action of an esterase that converts it into its pre-activated form. The dye is dark in its native state, but a photolysis reaction induced by 405 nm light causes the conversion of pre-activated DACT-1 into a bright red-fluorescent molecule, which also forms a cross-link with intracellular nucleophiles. These features ensure that DACT-1 can only be fully activated in the intracellular space, with cross-linking extending the stability of photolabeling. We synthesised DACT-1 in house, in EMBL's Chemical Synthesis Core Facility. In the case of experiments with RPE-1, IMR-90 and BJ-5ta cells, we used DACT-1 as a photolabel. In this case, nuclei were visualised with NucSpot Live 650 Nuclear Stain (Biotium). Both molecules were added to the cell culture medium at least three hours prior to the start of the photolabeling phase. Photolabeling for both H2B-Dendra2 and DACT-1 was performed by point scanning inside regions of interest covering the parental nucleus, with a 20X/0.8 NA air objective, 405 nm laser at 0.5% power, in the low intensity range, for 50 iterations.

In order to assess whether H2B-Dendra2 or DACT-1 would have an impact on the formation of nuclear atypia, we fixed and stained parental MCF10A cells, the MCF10A H2B-Dendra2 clone generated for this study, and MCF10A cells exposed to DACT-1 using Hoechst 33342. The frequency of cells containing micronuclei was quantified manually. No significant difference in micronucleus abundance is seen (**Supplementary Fig. 1f**), indicating there is no measurable impact of these dyes on nuclear atypia formation.

Microscope automation and imaging

MAGIC relies on full microscope automation and computer vision, to enable large-scale, phenotype-driven targeted photolabeling of single cells based on its Adaptive Feedback Microscopy loop. Adaptive-feedback microscopy can be understood as sequences of imaging and analysis jobs, where each step in the sequence depends on the execution and actionable information coming from the previous steps. This requires the controlled interaction between microscope hardware and computer vision algorithms to generate complex behaviours required for a fully automated experiment[7]. MAGIC automation leverages existing tools for building Adaptive Feedback Microscopy workflows, based on the *AutoMicTools* package for ImageJ (https://git.embl.de/halavaty/AutoMicTools) and a support Python library for the ZenBlue macro environment (https://git.embl.de/grp-almf/zeiss_zenblue_automation/). Additionally, we developed three main software components: the first component is a microscope control script that controls the automation workflow, integrates feedback information and directly controls the microscope hardware via Zeiss ZenBlue software. The second component, based on *AutoMicTools* package[8,9], manages image analysis tasks by interacting with the microscope control component, performing the analysis tasks and generating feedback files. The third component is a Python package called *magic_tools*, which we developed to extend the image analysis capabilities of our platform with ML; *magic_tools*

offers an image processing server, which runs on a cluster node and takes care of computationally intensive tasks such as the identification of micronuclei.

For our MAGIC experiments, we employed an LSM 900 laser scanning confocal microscope (Zeiss) equipped with additional widefield imaging capabilities. Microscope automation was implemented requiring two software components: a custom script for controlling the microscope and an ImageJ plugin to handle the communication between microscope and image analysis server. Control of the microscope was accomplished through the developer toolkit of the software ZEN, enabling control via scripts written in IronPython. We designed a custom script that implements the feedback loop as described in **Supplementary Fig. 2a**, moving between stage positions, triggering imaging experiments and coordinating between imaging and analysis operations. At the same time, image analysis tasks are accomplished via *AutoMicTools*[8], a Java library that works as an ImageJ plugin and coordinates with the microscope control script. For the purpose of this project, we additionally developed an *AutoMicTools* job class that handles image analysis via the *magic_tools* image processing server. The microscope control software and *AutoMicTools* interact by exchanging files in a "watchfolder" where images and JSON files are monitored to trigger acquisition or analysis jobs. Both custom software components are made available in the *magic_automation* repository.

The microscope control script iterates through several positions to perform three tasks: autofocusing, identifying micronuclei, and photoconverting target nuclei. Autofocus is accomplished by detecting the reflection signal of the glass dish bottom. To achieve this, the microscope acquires an XZ line scan with a 639 nm laser. The image is then detected and analysed by *AutoMicTools* to look for the location of highest intensity along the Z axis, representing the reflection of the glass. A feedback file is generated containing the coordinate of the best-focused slice, and this information is used to define the coordinates of the in-focus position for the next task. To identify micronuclei, we use a PlanApochromat 20X/0.8 NA M27 air objective to acquire an image Z-stack. This image consists of seven equidistant slices spanning a total of nine µm, centred on the focus coordinate identified in the previous step. *AutoMicTools* detects the image and sends it for analysis to an image analysis server based on *magic_tools*. The classification result is converted in polygon coordinates that define the contour of the nuclei to be photolabeled. Finally, for targeted photolabeling, the script parses the feedback file containing the polygon coordinates and defines regions of interest (ROIs) as in a bleaching experiment, before the experiment is triggered. Photolabeling is performed with a 405 nm laser scanning through the ROIs, for 50 iterations each, at speed 8 and 0.5% power. Two images before and after the photolabeling are acquired. After acquiring the confirmation image, the microscope proceeds to the next sample position for a new iteration.

During a typical experiment we define 200 fields of view per well, for a total of 800 positions, which are scanned over the course of 24 hours or until the desired yield is reached.

Online image analysis with *magic_tools*

MAGIC necessitates computer vision inferences to achieve online, on-the-fly image processing. Yet, the calculation of pixel and object-level features for phenotype classification is a computationally-intensive task that scales linearly with the number of pixels and objects to be evaluated as well as the number of features. Therefore, we developed *magic_tools* to enable parallelization of feature computation by means of an image processing server, and to support annotations and training of ML models.

To ensure an effective use of the platform, considering the potential range of future applications, we emphasise that it is unfeasible to create a model (or a collection of models) that covers every use case. With this requirement in mind, we opted for a pragmatic approach, focusing on gradient boosting classifiers which are simpler, but can work with a smaller training set and be quickly tailored for a specific experiment and deployed, while still achieving an appropriate performance for the experimental needs.

For on-the-fly detection of micronucleated cells, we integrated an image analysis pipeline into *magic_tools*, which includes a two-step semantic pixel segmentation and object classification based on extreme gradient boosting classifiers[10] trained on precomputed mathematical features. In the first step, we perform a maximum-intensity projection of the Z-stack and use an XGBoost classifier to perform a pixel semantic segmentation. For semantic segmentation, we compute pixel-level features by applying a series of Gaussian filters to capture information at different scales, and then filters for pixel intensity, edgeness and texture are calculated to generate the final feature outputs. An XGBoost classifier then generates a foreground prediction score for each pixel. Semantic segmentation is refined by morphological operations and on-demand watershed[11] to define nuclei objects. At this stage, micronuclei – if separated from the main nucleus – might form their own object or be part of a nuclear mask if they are adjacent to the nucleus. Therefore, we refine the nuclei masks to identify areas of convexity that deviate from the oval shape of a normal nucleus and thus capture micronuclei-candidate objects. We calculate object-level features for micronuclei candidates across multiple domains: basic descriptors, shape, intensity and texture. In addition, we calculate the same features for parental nuclei. Micronuclei and parent nuclei features are then concatenated by nearest-neighbour search, considering the shortest distance between a micronucleus and a nucleus edge. The concatenated feature vectors are then used as input for the second XGBoost classifier to identify micronuclei among all the candidate objects. Objects that cross a predefined probability threshold are considered eligible for targeted illumination and the parental nuclear masks are converted to polygon coordinates and packaged in a feedback file.

For parallelisation of feature computations, *magic_tools* uses two different approaches in the case of pixel or object-level features. In the case of pixel features, filter computations are parallelised on a pool of workers in two stages, first across Gaussians, then across feature filters. The final set of selected pixel features was determined according to their relative feature importance. Instead, in the case of object features, parallelisation occurs across objects, with all features calculated in sequence for each of them. This parallelisation enables inferences in the order of a few seconds, depending on the number of objects in the image. Another feature facilitating on-the-fly inference is the image processing server, which we deployed on a computational support node to leverage more computational power. The image processing server can receive images via a RESTful API, and we developed a custom *AutoMicTools* plugin to enable online image analysis by *magic_tools*.

Image analysis for normal nuclei detection utilises the same approach, but it uses a round-nucleus specific XGBoost classifier. In alternative, we selected cells with a low probability of carrying a micronucleus. All image analysis pipelines are available in the *magic_tools* repository.

XGBoost classifier training

XGBoost classifiers are employed in two crucial moments of the analysis pipeline: pixel semantic segmentation to detect foreground signal, and object classification to identify micronuclei and their parental nuclei. For semantic pixel segmentation, we performed a sparse annotation for foreground and background pixels in seven representative images, acquired with identical imaging settings used in all MAGIC experiments with a specific cell line. We then computed a set of pixel level features, as described above, to generate our ground truth set. We split the set for training (80%), validation (10%) and testing (10%) purposes. An XGBoost classifier was trained and hyperparameters were iteratively adjusted on the validation set. Finally, we assessed classifier performance on the test set.

For micronuclei classification in MCF10A, we manually annotated 200 random fields of view and integrated this with micronuclei candidate regions detected by image analysis. This resulted in the identification of more than 23,000 candidate micronuclei regions, of which 478 were ground truth micronuclei annotations. Object level features were calculated as described above (a complete list of features can be found in the repository). Also in this case, we split the set for training (80%),

validation (10%) and testing (10%) purposes, stratifying the splits by ground truth label. Model hyperparameters were fine-tuned on the validation set, to achieve at least 90% precision, while still recalling at minimum 50% of all micronuclei, for the entire image analysis pipeline – thereby striking an acceptable balance between sensitivity and specificity. Final classifier performance was assessed on the test set, taking into account the entire image analysis pipeline. To get a more objective measure of classifier performance, we performed several different dataset splits. The average precision-recall curves, with their standard deviation, were calculated from the aggregated data and presented in **Supplementary Fig. 2b**. We developed pixel and micronuclei classifiers tailored for the specific cell line models and to accommodate the different image settings, cell morphologies and experimental variation. Models are made available in the *magic_tools* repository.

Convolutional neural network based micronucleus classifier training

We additionally implemented a convolutional neural network for micronucleus classification, with the network architecture developed based on ConvNeXT (**Supplementary Table 3**)[12]. The training dataset consisted of fluorescence microscopy images cropped to 300×300 pixels. The image crops were centred either on a micronucleus or a non-micronucleus object and were generated with the image analysis pipeline for micronuclei detection described above. During training, image crops were augmented using random upsampling by a factor from 1.1 to 2.0, as well as rotation and random flipping. Images were rescaled to 128×128 prior to being utilised as an input to the network. We trained the network with a negative log likelihood loss function on the image class (L_{NLL}) and a negative log likelihood loss between the outputs of the network for two different data augmentations ($L_{consistency}$) to encourage rotation and scale invariance. The final weighted loss function of the network was $L = L_{NLL} + 0.1 \cdot L_{consistency}$. We trained the network with 5 independent initialization and train-test splits, with 15% of the data per class (micronucleus; no micronucleus) used to construct a withheld test set. Models and training scripts were implemented using Python 3.10, jax version 0.3.6 [13] and PyTorch version 1.11.0 [14].

Strandtools – an optimised single-cell CA calling algorithm for Strand-seq data

To detect CA in single-cell genomic data generated by Strand-seq, we developed a Python package called *strandtools* (https://git.embl.de/cosenza/strandtools), that improves on our previously developed tool MosaiCatcher[1], and is tailored for the specific task of handling CA detection in single cells and complex ploidy backgrounds. In Strand-seq data, the ratio in read counts in the W and C strand orientation states is related to the underlying number of template homologs, with specific ratios providing information on segmental copy-numbers and SV classes based on the principle of tri-channel processing (combining information on read depth, strand-orientation and haplotype phase)[1]. Particularly, strandtools leverages read coverage, as well as the ratio between W and C templates from multiple cells, to build Naive Bayes ML models and assign copy-number states in each single cell, including in scenarios with a non-disomic ploidy.

While an overview of the method is provided below, we anticipate a future publication elsewhere covering in detail all of the various features of *strandtools*. In particular, data processing uses read count tables generated by MosaiCatcher with a multi-step normalisation being performed as the first step[15]. The chromosomes are then segmented in regions of homogeneous coverage and strand orientation with a modified circular binary segmentation[16] algorithm. An OPTICS clustering algorithm[17] is then employed to identify clusters of homogeneous read count and W fraction, corresponding to combinations of templates and copy-number. Cluster labels are used as ground truth to train a naive Bayes classifier and generate a representation for copy-number assignment when the template ratio is informative. Then, the same genomic segments have the template ratio masked to train a second naive Bayes classifier to generate copy-number predictions for segments with homogeneous strand states (only W or only C reads). Copy-number assignment to the different clusters is solved by brute force, maximising the correlation between copy-number assigned to the

different clusters and their average read count. These two Naive Bayes classifiers are then applied to the entire data to generate copy-number predictions. Adjacent genomic segments with identical copy-number are iteratively merged into super-segments, which can be used for a second round of classifier training for better accuracy. Several quality control (QC) filters are applied to the predictions to generate the final copy-number probabilities: we exclude segments with uncertain predictions, a too high coefficient of variation as a measure of noise, or a too high number of low count chromosomal bins. Segments that are part of excluded genomic regions (e.g. centromeres) are likewise excluded. Finally, changepoints for copy-number breakpoints and SCEs are outputted. In our workflow, this output is used for detailed CA classification as described in the **Methods** section. The source code of *strandtools* is provided alongside this study (see **Code availability** section).

Code reproducibility

We provide a unified Docker container (https://git.embl.de/tweber/magic-container) that includes the complete MAGIC tool suite—*strandtools*, *magic_tools*, and *magic_automation*—along with all necessary dependencies such as ImageJ, JupyterLab, and FastAPI. The container supports three execution modes corresponding to key stages of the workflow: image pipeline development, Strand-seq data analysis, and full MAGIC experiment execution. In the latter mode, a browser-accessible GUI is provided for interactive configuration of ImageJ. A pre-built image has been made available to simplify deployment.

Computational sister cell pair discovery from Strand-seq data

To automatically detect sister cells, *strandtools* converts the strand state class into a numerical value: 1 for WW, 0 for WC and -1 for CC status. Then, the Pearson correlation coefficient between all pairs of cells in an experiment is computed. Sister cells are considered as cell pairs exhibiting a correlation coefficient below -0.9, which we find faithfully corresponds to sister cell pairs (**Supplementary Fig. 3f, Extended Data Fig. 1a**).

Sister cell pair analysis is not necessary for the discovery of *de novo* CAs with Strand-seq, but it can help reconstruct historical rearrangements happening during prior cell divisions. While we did not explicitly design our experiments to enrich for sister cells, we anticipate that in the future relatively straightforward adjustments to MAGIC could allow for such enrichment – for instance – synchronising cells and allowing them to divide after photolabeling, but before collection.

In addition, we note that the number of sister pairs captured might be influenced by the likelihood of cell division during the experiment: for example, we find higher numbers of sister cells in the spontaneous nuclei dataset (**Extended Data Fig. 1a**) as compared to targeted DSB induction (**Supplementary Fig. 3f**). This suggests that CRISPR treatment may impose additional stress on the cells, which could ultimately lead to more frequent cell cycle arrest (perhaps due to the sustained action of Cas9 resulting in DSBs), reducing the number of sister cells captured.

Designing single-guide RNA

Non-repetitive regions of 2–6 kb in size were identified within the centromeric and telomeric regions of chromosome 2q, which has a relatively low repeat content[18], as well as for chromosome 7q. For each site, we designed at least 10 single-guide RNAs (sgRNA) targeting these non-repetitive regions using the CRISPick tool (https://portals.broadinstitute.org/gppx/crispick/public), designing three sgRNAs per genomic region. On-target and off-target effect of sgRNAs were assessed by CRISPR-CUSTOM (https://eu.idtdna.com/site/order/designtool/index/CRISPR_CUSTOM) and Off-Spotter (https://cm.jefferson.edu/Off-Spotter/) tools. We aimed at an on-/off-target score of ≥50 (CRISPR-CUSTOM) and no binding per one, two, and three mismatches (Off-Spotter). The editing efficiency of the designed guides was assessed in sequence, using the TIDE assay[19]. For this purpose, two primers per sgRNA were designed by Primer-BLAST such that forward primer binds 150-300 bp

upstream of Cas9 cut site and that reverse primer binds 300-500 bp downstream of Cas9 cut site. Designed guides showed generally good efficiency. In any case, sgRNAs showing highest editing efficiency and corresponding PCR primers, which produced a single PCR product as well as high Sanger-seq quality, were chosen for downstream analyses.

Statistical testing for biases in CA frequencies, SCEs and breakpoint locations

Binomial testing for chromosome bias in CA landscapes. To determine non-random bias in chromosomes affected by CAs, we first derived the expected probability of having any chromosome hit by a CA by dividing the number of annotated CAs and the total number of chromosomes in MCF10A or RPE-1 cells. From this, under the assumption that any chromosome would be equally affected by a CA, we derived a binomial distribution for the different chromosomes depending on their consensus copy-number. We executed binomial tests for each chromosome, considering the observed CA count as the number of successful trials. Finally, P-values were adjusted by the Benjamini/Hochberg method.

Permutation testing. Breakpoint locations of SCEs and copy-number changes were detected using *strandtools*. To test for breakpoint enrichment in fragile sites, early and late replicating regions, we performed a permutation test utilising the *regioneR* package available in Bioconductor. Breakpoint regions were permuted with the *randomizeRegions* function 10,000 times. As evaluation functions, we employed the number of overlaps (*numOverlaps*) for fragile sites, early and late replicating regions, while for G4 quadruplexes we summed their occurrence in the permuted bins affected by a breakpoint. P-values were adjusted by the Benjamini/Hochberg method. Fragile site locations were obtained from HumCFS database[20], and G4 quadruplex data was obtained from a previous study[21]. We find no CA enrichment for BrdU fragile sites, excluding the possibility of BrdU acting as common CA trigger – consistent with prior works reporting that the low concentration BrdU pulses utilised in Strand-seq do not enhance genetic instability[22].

To determine enrichment for early and late replicating regions, we employed the pre-processed UW Repli-seq tracks available on the USCS Genome Browser database[23]. The data was remapped to the hg38 genome using the UCSC LiftOver tool, binned in 200 kbps windows and aggregated over the different cell lines. We calculated the mean replication timing score and its standard deviation for all genomic bins. In order to filter regions replicating early or late across cell lines, we set a low standard deviation (score standard deviation =< 5) as requirement, and then assigned bins to replicating early or late replicating regions depending on their average score (early =< 20, late >=60). Permutation tests for chromosome size took into account consensus copy-numbers for each chromosome. CA annotations – regardless of the specific CA class – were permuted over all chromosomes in the dataset. For the test statistic, CA counts per chromosome were aggregated and correlation to chromosome length was computed using the Pearson correlation coefficient. P-values were then calculated from our observations, and the permuted distribution derived from 50,000 iterations.

Binomial testing for coordinated Watson(W)/Crick(C) gains. To test for the coordinated amplification of W and C templates, we focused on gains with an increment of exactly two. If the two extra segments were inherited independently, then their expected probability in a daughter cell would be 0.25 W/W, 0.50 W/C, and 0.25 C/C, respectively[1]. By considering every cell division as its own Bernoulli trial, and considering the W/C outcome as 'success', we employed a binomial test to examine if the observed data agrees with this model. In this case the probability of success for a trial is 0.5, the number of trials corresponds to the number of cells carrying an amplification, and the number of successes is the number of cases where we observe a WC 'coordinated gain'. Among 18 inferred acentric gains with a copy-number increment of 2, the W/C ratio remains 1:1 in all 18 cases, based on which we reject the hypothesis that the fragment gains segregate independently (P<7.63e-06). We thus infer based on the single–cell genomic data analysed that the amplified

segments are part of the same derivative chromosome, where they are arranged in an inverted orientation.

CA rate estimation by bound-constrained minimization

We simulated a population of cells, allowing each cell to progress through mitosis and, upon division, acquire a new nuclear phenotype and *de novo* CAs according to the model's transition probabilities. At the end of the simulation, we measured the error between the inferred CA rate and the empirically observed CA frequency in normal and micronucleated cells. An optimization algorithm minimising this error allows estimation of the basal CA rate.

To estimate the CA rates *R*, we performed a bound-constrained minimization of the sum of squared errors between simulated and target *de novo* CA frequencies. To achieve this, we employed a modification of the "Powell" method[24]. This optimization algorithm searches the parameter space by doing a sequential, bi-directional minimization for each search vector, until a local minimum of the function is found. As rates *R* are probabilities of an event happening, we bound these values between 0 and 1. For each estimation, we ran 50 optimization rounds, initialising the *R* parameters from the same set of uniformly random numbers. The output rates of one estimation were computed by the average of the 50 optimization outputs, weighed by the sum of squared errors.

CA events detected in a cell population reflect a combination of newly arisen CAs and those inherited from earlier cell divisions, thus not all observed CAs can be considered *de novo*. Under this hypothesis, we adjusted the target CA frequencies by subtracting a scalar value from the observed CA count in both normal and micronucleated cells. We performed first a sparse search, then focused on the following ranges for normal cells: 5 to 19% for WT and 18 to 34% for *TP53-/-* models. We thus derived CA rate estimates for each of the adjusted frequency values with their corresponding sum of squared errors values. We used this latter value as a measure of goodness-of-fit to judge how well the model fitted the observations. After applying this adjustment, the optimal fit for normally nucleated cells corresponds to a 10% *de novo* CA proportion for WT and 21% for *TP53-/-* cells (**Supplementary Fig. 6c**). With applying this adjustment parameter to micronucleated cells, we obtain a *de novo* CA proportion of 47% in WT and 72% in *TP53-/-* conditions (**Supplementary Fig. 6d-e**). Final CA rates were determined and reported in **Fig. 3h**.

Western blotting

Western blotting to validate p53 knock-out status of cell lines was performed as follows: a pellet from 2 million cells was resuspended in lysis buffer (1X RIPA buffer Cell Signaling, 1X protease inhibitor cocktail Roche, 1 mM PMSF Roth) and incubated on ice for 10 minutes. Debris was pelleted and the supernatant was frozen for 2 hours at -80 °C to assist lysis. 40 µg of protein were mixed with loading dye and heated for denaturation. Samples were loaded onto a precast 4-15% Tris-Glycine gel (Bio-Rad) and run in 1X Laemmli buffer at 100V for 10 minutes, followed by 180V for 45 minutes. We then performed semi-dry transfer onto a nitrocellulose membrane with a Trans-Blot Turbo Transfer System. After transfer, the membrane was blocked with 5% milk in TBS-T and primary antibodies (1:200 p53, DO-1 mouse mAb, Santa Cruz; 1:1000 nucleolin, D4C7O rabbit mAb, Cell Signaling) were incubated for 2 hours in blocking buffer. The membrane was washed three times in TBS-T, and an HRP-coupled secondary antibody (goat anti-mouse IgG-HRP 1:10000, Invitrogen) was incubated for 45 minutes. After three final washes, the membrane was incubated in ECL substrate (Bio-Rad) for 1-3 minutes at room temperature in the dark. Protein bands were visualised using a GelDoc XR Imaging system.

Supplementary Notes

Limitations in detection of micronuclei

To investigate possible biases and limitations in the detection of micronuclei by the classification models, we utilized SHAP (SHapley Additive exPlanations): a concept from cooperative game theory applied in machine learning to estimate the weight of each input feature in determining a model's output[25]. By looking at the SHAP values difference between false negatives and true positives, we could identify features driving discrepancies between these, as a quantitative fairness metric to investigate model bias. The top 5 features identified are highly correlated with each other and imply the presence of three primary factors characterizing the false negatives cases, which we attribute to segmentation quality, object size and object adjacency (**Supplementary Fig. 11b,c**):

Segmentation Quality: Poor segmentation would affect features such as roundness and Hu moments, which capture the 'round' shape of a micronucleus. We validated this by annotating cases of poorly segmented masks, where portions of the nucleus were incorrectly included. We observed that, under equal conditions, recall was significantly higher for correctly segmented micronuclei (**Supplementary Fig. 11d**). As a result, segmentation-related artifacts can elevate feature values in poorly segmented instances (**Supplementary Fig. 11h**).

Object Size: Features like area-perimeter ratio and magnitude of the object long axis directly correlate with object size. We noticed that model performance decreases for extreme cases, particularly for smaller micronuclei (**Supplementary Fig. 11e**). Subdividing the dataset confirmed that feature values associated with object size were significantly lower in false negatives, tracking their smaller size (**Supplementary Fig. 11i**). Additionally, fluorescence intensity was dimmer in these cases, emphasizing that dim, small micronuclei are inherently challenging to detect (**Supplementary Fig. 11j**), especially when the dataset is dominated by similarly-sized, small objects like debris (**Supplementary Fig. 11k**). We emphasise that challenges associated with detecting very small micronuclei in datasets crowded with small, similarly-sized objects like debris are comparable for both manual annotation and machine learning classification methods.

Object adjacency: Objects that are adjacent to the main nucleus pose a difficult challenge in terms of segmentation, compared to those that exist in isolation. The hist_ng0_mni feature measures the lowest bin intensity in the immediate surrounding of the micronucleus, sensing if this is adjacent to the parental nucleus or isolated (**Supplementary Fig. 11l**). This difference, similarly to other segmentation quality issues, can be reflected in a relatively lower recall for these micronuclei (**Supplementary Fig. 11f**).

Confirmation of the role of these three factors in driving the group differences in false negatives and true positives is given by the fact that filtering the dataset based on adjacency to the nucleus, object size or segmentation quality decreases the group difference for the corresponding biased features (**Supplementary Fig. 11m**).

While these factors provide important insights into model limitations, we stress that they are unlikely to affect our key findings. False negatives from poorly segmented masks are expected, but reflect technical challenges in segmentation and do not systematically impact micronucleus features central to our analysis. The lower recall for adjacent micronuclei is also connected to difficulty in segmentation of micronuclei candidates. However, micronuclei are mobile, shifting between isolated and adjacent positions, reducing the potential for consistent bias. Detection challenges near the microscope's resolution limit, particularly for small or dim micronuclei with low signal-to-noise ratios, are also of technical nature and inherent to the imaging process. In our case, the wide dynamic range of marker fluorescence can complicate simultaneous detection of dim and bright objects, but this reflects expression levels of the transfected marker rather than biologically relevant signals. Moreover, small, dim micronuclei are difficult to annotate even for human experts, and the intrinsic uncertainty of this detection might be reflected in the model.

Differential expression analysis of micronucleated cells with MAGIC

By coupling MAGIC to SMART-seq2,[26] we performed comparative single-cell RNA sequencing (scRNA-seq) analysis on micronucleated and normal cells in the MCF10A and RPE-1 cell lines. Cells were screened and photolabeled using the MAGIC platform, then directly sorted as single cells in lysis buffer and processed as previously described[26]. Briefly, single-cell sequencing reads were aligned to the reference genome (GRCh38) using STAR (version 2.7.11b/GCC-13.2.0, https://github.com/alexdobin/STAR), and gene count matrices were generated with HTSeq (version HTSeq/2.0.2, https://github.com/htseq/htseq). Quality control, normalization, and differential expression analysis were conducted using the *edgeR* package (version 4.4.0, https://bioconductor.org/packages/release/bioc/html/edgeR.html). Gene set enrichment analysis was performed with the Fast Gene Set Enrichment Analysis (*fgsea*) (version 1.32.0, https://bioconductor.org/packages/release/bioc/html/fgsea.html) using the "Hallmark" and "Gene Ontology" (GO) predefined gene sets from MSigDB (https://www.gsea-msigdb.org/gsea/msigdb). For the GO enrichment analysis, we simplified results using *rrvgo* (version 1.18.0, https://www.bioconductor.org/packages/release/bioc/html/rrvgo.html), which reduces redundancy based on semantic similarity. All analyses were carried out in R (version 4.4.2) following standard bioinformatics workflows.
In total, we analysed 170 micronucleated and 76 normal cells from MCF10A, and 84 micronucleated and 71 normal cells from RPE-1 (**Supplementary Tables 11-16, Extended Data Fig. 7a**). We observe a somewhat higher quality for the newly generated MCF-10A dataset, resulting in the identification of a higher number of differentially expressed (DE) genes (**Extended Data Fig. 7a**). Nonetheless, data quality was acceptable in both scRNA-seq experiments, allowing us to infer DE genes using *fgsea* – revealing enriched pathways from the "hallmark collection" of the Human Molecular Signatures Database (MSigDB), where each gene set represents specific well-defined biological states[27].

This analysis provided additional insights into the effects of spontaneous micronucleation in these cells: We observe shared suppression of proliferation markers in both MCF10A and RPE-1 for micronucleated cells, as indicated by the downregulation of hallmark gene sets such as E2F_TARGETS, G2M_CHECKPOINT, and MYC_TARGETS (V1 and V2) (**Extended Data Fig. 7b**). This finding, notably, aligns with our live-cell imaging based data, which show a cell cycle arrest in micronucleated cells (**Supplementary Fig. 1d**). In MCF10A, we detect activation of the P53_PATHWAY hallmark gene set (**Extended Data Fig. 7b**), consistent with p53-driven cell cycle arrest. This finding is further bolstered by the lack of cycle arrest during live-cell imaging of *TP53-/-* models following micronucleation (**Supplementary Fig. 4g,h**). We also observe significant upregulation of *TP53* (logFC: 1.655, adj. p-value: 1.439e-16) in micronucleated RPE-1 cells, implying a similar process is at play in these cells.

Exposure of micronuclei content to cytoplasm has previously been reported to activate the cGAS-STING pathway, leading to NF-κB activation and the expression of IFN-gamma response genes and IL-6.[28] In accordance with this, our pathway enrichment results include activation of INTERFERON_GAMMA_RESPONSE, IL6_JAK_STAT_SIGNALING, and TNFA_SIGNALING_VIA_NFKB pathways in MCF10A cells (**Extended Data Fig. 7b**). GO term enrichment analysis further corroborates these findings, emphasizing suppression of proliferation and metabolism, particularly through the downregulation of genes related to DNA replication and chromosome segregation (**Extended Data Fig. 7c**). Cell migration pathways are uniquely enriched in MCF10A, suggesting additional biological processes specific to this cell line (**Extended Data Fig. 7c**).

Pan-cancer WGS cohorts

Data from the Pan-Cancer Analysis of Whole Genomes (PCAWG) Consortium[29], encompassing 2,583 donors across 47 tumour types, was downloaded from the International Cancer Genome Consortium

(ICGC) Data Portal (https://dcc.icgc.org/releases/PCAWG, **Supplementary Table 4**). To avoid redundancies due to multiple aliquots originating from the same donor[29], we selected one representative aliquot per donor (as defined in "aliquot_donor_tumor.whitelist.tsv.gz" in the ICGC Data Portal). For the copy-number (CN) data, we merged consecutive segments that had the same copy-number (CN) value.

To comprehensively infer isochromosomes across diverse cancer types, we combined CN data from PCAWG[29] and from a separate TCGA pan-cancer WGS resource, the latter comprising data from 4,957 tumour-normal pairs encompassing 30 tumour types (https://portal.gdc.cancer.gov/). For TCGA, WGS datasets with an average read length of ≥100 bp and passing the following quality control (QC) criteria were considered for our analyses. QC was conducted using AMBER and PURPLE, with samples discarded if at least one of the following conditions was met: (1) FAIL_CONTAMINATION, where tumour contamination in homozygous sites from the normal sample exceeded 10%; and (2) FAIL_NO_TUMOUR, where no evidence of tumour was found in the sample. For the 698 donors that were found in both the TCGA and PCAWG cohorts, only the TCGA sample was kept for isochromosome analysis (n=1,985 for PCAWG; n=4,957 for TCGA). Raw sequencing reads were mapped to the GRCh38 build of the human reference genome using BWA-MEM (v0.7.17-r1188).[30] Aligned reads were processed following the GATK Best Practices workflow (v4.1.8.0) to remove duplicates and recalibrate base quality scores[31]. Germline and somatic SNVs were called and filtered using SAGE (v2.8). Somatic SVs were detected using GRIDSS2 (v2.12.0, available at https://github.com/PapenfussLab/gridss)[32] and filtered using GRIPSS (v1.9). The B-allele frequency (BAF) of heterozygous SNPs was computed with AMBER (v3.5) and read depth ratios were calculated using COBALT (v1.11). B-allele frequency, read depth information, breakpoint coordinates and single nucleotide variant (SNV) allele frequencies were integrated to estimate somatic copy-number aberrations using PURPLE (v2.54).[33] The raw copy-number values estimated for each segment were rounded to their nearest integer for further analysis. Then, consecutive CN segments that had the same CN value were merged. Only copy-number values inferred by PURPLE using BAF data were considered for downstream analysis. SAGE, GRIPSS, AMBER, COBALT, PURPLE and LINX were developed by the Hartwig Medical Foundation (HMF)[33] and are freely available on GitHub at https://github.com/hartwigmedical/hmftools.

Comparing copy-number features from spontaneous micronuclei with the PCAWG resource

Extracting copy-number features for abnormal chromosomes

We compared CN features between MCF10A and the PCAWG WGS dataset[29] by adapting methodology from Drews and colleagues[34] (*i.e.*, the CINSignatureQuantification package; https://github.com/markowetzlab/CINSignatureQuantification). Whereas the original approach from Drews et al. was designed for the analysis of bulk whole-genome sequencing data of samples harboring high chromosomal instability (CIN), we devised adaptations to this approach to facilitate the comparison between bulk data of highly evolved cancer samples from PCAWG and single-cell data from spontaneously micronucleated MCF-10A.

The method of Drews and colleagues quantifies five CN features: (1) length of non-diploid segments; (2) number of breakpoints per 10 Mb; (3) number of breakpoints per arm; (4) number of CN oscillations; and (5) magnitude of the changepoints[34]. It then computes CIN signatures based on these five features. By default, only samples with high CIN are included in the analysis by filtering out samples with fewer than 20 CAs (DCIN = 20 in the removeQuietSamples() function). We lowered this threshold to DCIN = 2 (lowest possible value within the package framework) to account for our early-stage MCF-10A samples harbouring few CAs, which would otherwise have been filtered out.

We noticed that, in largely uneventful samples, the CIN signal from chromosomes harbouring CAs could become "diluted" among all of the other chromosomes harbouring few or no CAs. We addressed this by pursuing the analysis of CIN signal per chromosome, instead of per sample or per cell. Furthermore, we determined that performing the analysis at the feature level, instead of at the signature level, was not only sufficient for comparing CIN signals between chromosomes, but also improved the interpretability of the results because each feature directly represents a biological signal. To perform the analysis at the feature level, for each chromosome we averaged the log2-transformed values (adding a pseudocount of 1) of each feature. Then, for each feature we normalised the values across all chromosomes between 0 and 1. We applied this normalization separately to three datasets of interest – MCF10A, PCAWG, as well as both combined (MCF10A + PCAWG). As input, we provided the per-chromosome CN data as integer values. For MCF-10A, we modified the input CN data by computing max(0, CN - consensus + 2), so that non-diploid segments whose CN is the same as the consensus are not considered to be CAs. Further minor adaptations introduced to CINSignatureQuantification included skipping the smoothing of segments by smoothSegments() when the input data only has one segment, and correcting an unwanted behaviour during the computation of CN oscillations by the getOscillationDrews() function, which in its original form did not count oscillations if they reached until the end of the chromosome.

We performed hierarchical clustering of the averaged and normalized CIN features using Euclidean distances and ward.D2 linkage as implemented in the hclust() function in R. For each heatmap, we determined the optimal number of clusters using the gap statistic as implemented in cluster::clusGap() combined with factoextra::hcut(). Then, we plotted the heatmaps using the ComplexHeatmap package. For plotting purposes, we applied 99% winsorization to the mean log2-transformed feature values prior to normalisation. In the combined clustering of PCAWG and MCF10A chromosomes, we assessed the enrichment or depletion of each cluster in MCF10A chromosomes by permuting the cluster labels 1000 times and comparing the observed proportion of MCF10A chromosomes in each cluster with the distribution of simulated values. Finally, we corrected the P-values for multiple testing using the Benjamini-Hochberg method.

We note that, although our adaptations allowed us to investigate similarities in CIN features between spontaneously micronucleated MCF-10A and highly evolved PCAWG tumours, challenges persist in applying CIN signature methodologies to early- vs. late-stage samples. In particular, discrete CAs may overlap one another over time, potentially masking their mechanistic origin, especially in advanced tumours[34,35]. This masking can be exacerbated in bulk sequencing data, which often reflects a mixture of different sets of cells harbouring different combinations of CAs. Finally, experimental validation linking CIN signatures with putative aetiologies is currently still limited. With this in mind, our approach represents a first step towards the application of CIN feature analysis to relatively simple CA patterns.

PCAWG based copy-number pattern cluster enrichment analysis

To assess similarities between the CA patterns in MCF10A and those observed in the primary cancer genomes[29], we utilised the methodology adapted from[34] as described above. For each chromosome that had at least two non-diploid segments, we computed five features related to non-diploid segment length, number of CN breakpoints per 10 Mb, number of CN breakpoints per arm, number of CN oscillations, and magnitude of CN changepoints. Next, we performed a joint clustering of these features computed on PCAWG and MCF10A chromosomes (**Supplementary Fig. 9**). We find that MCF10A chromosomes cluster together with PCAWG chromosomes, leading to five major clusters.

Cluster 1 is characterised by relatively simple chromosomes, harbouring few breakpoints and long segments. Cluster 2 contains more complex chromosomes than cluster 1, harbouring comparatively shorter segments and higher breakpoint density, as well as medium/long chains of oscillations. Cluster 3 contains highly complex chromosomes, harbouring short non-diploid segments, the highest breakpoint density, and the highest magnitude of CN changepoints, including short copy-number oscillations. Cluster 4 is characterised on average by the longest chains of oscillations and the shortest

non-diploid segments, but breakpoint density is moderate. Finally, cluster 5 contains chromosomes with medium/short non-diploid segments, medium/high breakpoint density, and medium/short chains of oscillations.

The distribution of MCF10A chromosomes among clusters is not uniform, with clusters 1 and 2 enriched in MCF10A chromosomes, whereas clusters 3, 4, and 5 are depleted (permutation test, FDR < 0.05). Given that clusters 1 and 2 are characterised by relatively large size copy-number alterations (CNAs) and few breakpoints, we infer that these are likely to represent early stages of chromosome evolution, acquired in one or few cell cycles. Overall, the four clusters that included at least one MCF10A chromosome contained 91% of all analysed PCAWG chromosomes, with the two clusters that are enriched in MCF10A chromosomes containing 43% of all analysed PCAWG chromosomes. The remaining clusters, by comparison, are likely to represent later stages of somatic karyotype evolution in cancer or, in alternative, they could be generated by other processes. In this regard, we note that cluster 4 is characterised by CNAs of less than 200 kb on average – too small to be reliably detectable using Strand-seq[1]. Clusters 3 and 5, by comparison, are characterised by a high number of CN states, indicative of extensive chromosome remodelling across a larger number of cell divisions in combination with Darwinian selection[29] (**Supplementary Fig. 10a**). From these comparisons, we conclude that while there are some remaining challenges in applying current CNA signature methodologies to CA patterns at early stages of tumour evolution, the *de novo* CA patterns we document in association with spontaneous micronucleation are generally compatible with those seen in tumour genomes.

Quantification of aneuploidies in the PCAWG resource

Quantification of whole chromosome aneuploidies. To quantify whole-chromosome gains and losses in primary cancer genomes, we leveraged the PCAWG CN calls[29] (available at https://dcc.icgc.org/api/v1/download?fn=/PCAWG/consensus_cnv). We limited our analysis to chromosomes where the only event was a whole-chromosome gain or loss, and no further copy-number alterations (CNAs) were present. To this end, we searched for chromosomes that: (1) had only one copy-number segment; and (2) had a copy-number value above or below the ploidy of the aliquot[29] (whole-chromosome gain or loss, respectively), regardless of the magnitude of the difference. We defined the ploidy of each aliquot as the most frequent copy-number state across all genomic bases that had copy-number information. We called all chromosomes that passed this first set of filters our "unfiltered" dataset. Notably, in our unfiltered dataset, 3582/5139 (69.7%) events represent whole-chromosome losses (**Extended Data Fig. 9a**). This observation is in line with previous reports, which predict a bias of whole-chromosome losses over gains in cancer[36].

In some aliquots, ploidy could not be unambiguously assigned to an integer number. To exclude these aliquots from the analysis, we filtered out those in which the number of genomic bases affected by the most frequent copy-number state was less than three times larger than the number of genomic bases affected by the second most frequent copy-number state. In this dataset, 1787/2215 (80.7%) whole-chromosome events are losses (**Extended Data Fig. 9a**). As an alternative approach, we excluded all PCAWG aliquots that exhibited a whole-genome duplication (WGD) event, as inferred previously[37] for the PCAWG resource. Notably, also in this non-WGD dataset, the vast majority of *de novo* whole chromosome alteration events (1645/2017; 81.5%) represent chromosome losses, with chromosomal gains being comparably rare.

Analysis of 7q losses in breast cancer. To assess the frequency of chromosome 7q losses in breast cancer, we reanalyzed copy-number data of PCAWG representative aliquots classified as "Breast-AdenoCA" (n = 195), "Breast-LobularCA" (n = 13), or "Breast-DCIS" (n = 3). We considered chromosome 7 to have a q-arm loss if >80% of the q-arm had a CN value below the inferred ploidy of the aliquot. This restricted the analysis to 78 breast cancer aliquots that had non-ambiguous ploidy, out of which 4 (5.1%) had 7q losses (**Supplementary Fig. 10c**). Relaxing the

filter of % of base positions with lower CN to a more permissive 50% led to only two additional hits (6/78, 7.7%), and they were compatible with terminal losses as well as with extensive chromothripsis.

Inference of isochromosomes using bulk WGS data

Methodological approach. To infer a highly curated set of likely isochromosomes in human cancer cohorts, we designed a restrictive set of criteria that prioritised specificity over sensitivity. We performed the search in both the PCAWG and TCGA datasets, omitting 698 PCAWG donors that were redundant with TCGA (N = 1985 for PCAWG; N = 4957 for TCGA, combined N = 6942) (**Supplementary Table 5**). We excluded sex chromosomes and acrocentric chromosomes from this analysis. To prepare the data for the isochromosome search, first we smoothened the CN profiles by removing segments smaller than 10 kb and merging consecutive segments with the same CN state. Then, we excluded chromosomes with more than 150 CAs to filter out highly rearranged chromosomes. Next, we searched for putative isochromosome-like structures as follows. For the detection of putative isochromosomes of the q-arm, we conducted the search from the beginning of the chromosome to the centromere, while for the p-arm, we conducted the search from the end of the chromosome towards the centromere. First, we searched each chromosome arm for CN changepoints where the minor CN transitions from 0 to a non-zero CN state, only considering changepoints that mapped closer to the centromere than to the telomere. Next, for each CN changepoint, we estimated the modal CN state upstream and downstream of the changepoint for both the minor and the total CN. Finally, for each identified CN changepoint, we applied the following criteria to identify isochromosome-like structures:

a. More than 90% of the nucleotide positions both upstream and downstream of the changepoint have a CN equal to the modal CN value upstream or downstream of the changepoint, respectively.
b. The modal minor CN value is 0 upstream of the changepoint and different from 0 downstream.
c. Downstream of the changepoint, the modal total CN is at least 2 units greater than the modal minor CN.
d. The modal total CN downstream the changepoint is at least 2 units greater than the upstream total CN, and the difference is a multiple of 2.
e. Either (i) the modal total CN upstream of the changepoint is equal to the modal minor CN downstream, or (ii) the sum of the modal total CN upstream of the changepoint and the modal minor CN downstream equals the modal total CN downstream.
f. No more than 2 CAs occur between the changepoint and the centromere, and fewer than 30 CAs occur downstream of the changepoint.

If more than one changepoint satisfied the criteria above for a given chromosome, we selected the changepoint such that the highest percentage of nucleotide positions both upstream and downstream of the changepoint had a CN value equal to the upstream or downstream modal CN, respectively.

Quantification of putative isochromosomes and centromere-breakpoint distances. We inferred 1,060 putative isochromosomes (344 in PCAWG and 716 in TCGA; **Supplementary Table 6**) using this approach. Out of the cohorts that have more than 25 donors, the most frequent isochromosome after normalising by cohort size is i(17q) in PBCA-DE (48/230, 20.9%) (**Supplementary Fig. 10b**). The PBCA-DE cohort includes paediatric brain cancers such as medulloblastoma, in which highly recurrent i(17q) events have been previously described as highly recurrent[38]. The second most recurrent event is i(8q) in hepatocellular carcinoma (LIRI-JP: 32/250, 12.8%), followed by i(17q) in colon adenocarcinoma (TCGA-COAD: 16/127, 12.6%) and i(8q) in head and neck squamous cancer (TCGA-HNSC: 51/432, 11.8%). Overall, the most frequent isochromosomes across cohorts are i(8q) (n = 238), i(17q) (n = 210), i(3q) (n = 108), and i(5p) (n = 96), all of which have been previously

described as highly recurrent[38]. In conclusion, our method, reassuringly, retrieves previously described recurrent isochromosomes.

Encouraged by these observations, we assessed the distances between the inferred isochromosome internal breakpoint and the centromere. The proportion of inferred isochromosomes that have the breakpoint outside the centromere, and thus are likely to be dicentric, is 55.2% in PCAWG and 30.7% in TCGA (**Extended Data Fig. 9b**). The difference in proportions could be due to differences in assembly (hg19 in PCAWG, hg38 in TCGA), cancer type compositions of each cohort, and data quality, thus making it difficult to compare results between cohorts. It is reassuring to note that for the inferred dicentric isochromosomes, the median distance between the centromere and the internal breakpoint is 3.2 Mb in PCAWG (IQR = 1.9-6.6 Mb) and 2.9 Mb in TCGA (IQR = 1.1-6.1 Mb) (**Extended Data Fig. 9c**), showing good agreement across the two cohorts. In addition, the distribution of distances extends up to 37 Mb in PCAWG and 42 Mb in TCGA, with centromere-breakpoint distances of approximately 20 Mb observed commonly. In summary, our results suggest a heterogeneous distribution of isochromosome changepoints, with a substantial proportion of them being megabases away from the centromere.

References

1. Sanders, A. D. *et al.* Single-cell analysis of structural variations and complex rearrangements with tri-channel processing. *Nat. Biotechnol.* **38**, 343–354 (2020).
2. Gomes, A. M. *et al.* Micronuclei from misaligned chromosomes that satisfy the spindle assembly checkpoint in cancer cells. *Curr. Biol.* **32**, 4240–4254.e5 (2022).
3. Cortés-Ciriano, I. *et al.* Comprehensive analysis of chromothripsis in 2,658 human cancers using whole-genome sequencing. *Nat. Genet.* **52**, 331–341 (2020).
4. Gurskaya, N. G. *et al.* Engineering of a monomeric green-to-red photoactivatable fluorescent protein induced by blue light. *Nat. Biotechnol.* **24**, 461–465 (2006).
5. Récamier, V. *et al.* Single cell correlation fractal dimension of chromatin: a framework to interpret 3D single molecule super-resolution: A framework to interpret 3D single molecule super-resolution. *Nucleus* **5**, 75–84 (2014).
6. Halabi, E. A. *et al.* Dual-Activatable Cell Tracker for Controlled and Prolonged Single-Cell Labeling. *ACS Chem. Biol.* **15**, 1613–1620 (2020).
7. Morgado, L., Gómez-de-Mariscal, E., Heil, H. S. & Henriques, R. The rise of data-driven microscopy powered by machine learning. *J. Microsc.* **295**, 85–92 (2024).
8. Fuqua, T. *et al.* Dense and pleiotropic regulatory information in a developmental enhancer. *Nature* **587**, 235–239 (2020).
9. Fuqua, T. *et al.* An open-source semi-automated robotics pipeline for embryo immunohistochemistry. *Sci. Rep.* **11**, 10314 (2021).
10. Chen, T. & Guestrin, C. XGBoost: A Scalable Tree Boosting System. *arXiv [cs.LG]* (2016).
11. Abdolhoseini, M., Kluge, M. G., Walker, F. R. & Johnson, S. J. Segmentation of Heavily Clustered Nuclei from Histopathological Images. *Sci. Rep.* **9**, 4551 (2019).
12. Liu, Z. *et al.* A ConvNet for the 2020s. *arXiv [cs.CV]* (2022).
13. Bradbury, J. *et al.* *JAX: Composable Transformations of PYthon+NumPy Programs*. (2018).
14. Paszke, A. *et al.* PyTorch: An imperative style, high-performance deep learning library. *Adv. Neural Inf. Process. Syst.* **abs/1912.01703**, (2019).

15. Weber, T., Cosenza, M. R. & Korbel, J. MosaiCatcher v2: a single-cell structural variations detection and analysis reference framework based on Strand-seq. *Bioinformatics* **39**, (2023).

16. Olshen, A. B., Venkatraman, E. S., Lucito, R. & Wigler, M. Circular binary segmentation for the analysis of array-based DNA copy number data. *Biostatistics* **5**, 557–572 (2004).

17. Ankerst, M., Breunig, M. M., Kriegel, H.-P. & Sander, J. OPTICS: ordering points to identify the clustering structure. *SIGMOD Rec.* **28**, 49–60 (1999).

18. Hillier, L. W. *et al.* Generation and annotation of the DNA sequences of human chromosomes 2 and 4. *Nature* **434**, 724–731 (2005).

19. Brinkman, E. K., Chen, T., Amendola, M. & van Steensel, B. Easy quantitative assessment of genome editing by sequence trace decomposition. *Nucleic Acids Res.* **42**, e168 (2014).

20. Kumar, R. *et al.* HumCFS: a database of fragile sites in human chromosomes. *BMC Genomics* **19**, 985 (2019).

21. Marsico, G. *et al.* Whole genome experimental maps of DNA G-quadruplexes in multiple species. *Nucleic Acids Res.* **47**, 3862–3874 (2019).

22. van Wietmarschen, N. & Lansdorp, P. M. Bromodeoxyuridine does not contribute to sister chromatid exchange events in normal or Bloom syndrome cells. *Nucleic Acids Res.* **44**, 6787–6793 (2016).

23. Hansen, R. S. *et al.* Sequencing newly replicated DNA reveals widespread plasticity in human replication timing. *Proc. Natl. Acad. Sci. U. S. A.* **107**, 139–144 (2010).

24. Powell, M. J. D. An efficient method for finding the minimum of a function of several variables without calculating derivatives. *Comput. J.* **7**, 155–162 (1964).

25. Lundberg, S. M. *et al.* From local explanations to global understanding with explainable AI for trees. *Nat. Mach. Intell.* **2**, 56–67 (2020).

26. Picelli, S. *et al.* Full-length RNA-seq from single cells using Smart-seq2. *Nat. Protoc.* **9**, 171–181 (2014).

27. Liberzon, A. *et al.* The Molecular Signatures Database (MSigDB) hallmark gene set collection. *Cell Syst.* **1**, 417–425 (2015).

28. Chen, Q., Sun, L. & Chen, Z. J. Regulation and function of the cGAS-STING pathway of

cytosolic DNA sensing. *Nat. Immunol.* **17**, 1142–1149 (2016).

29. ICGC/TCGA Pan-Cancer Analysis of Whole Genomes Consortium. Pan-cancer analysis of whole genomes. *Nature* **578**, 82–93 (2020).
30. Li, H. & Durbin, R. Fast and accurate short read alignment with Burrows-Wheeler transform. *Bioinformatics* **25**, 1754–1760 (2009).
31. van der Auwera, G. & O'Connor, B. D. *Genomics in the Cloud: Using Docker, GATK, and WDL in Terra*. (O'Reilly Media, Incorporated, 2020).
32. Cameron, D. L. *et al.* GRIDSS2: comprehensive characterisation of somatic structural variation using single breakend variants and structural variant phasing. *Genome Biol.* **22**, 202 (2021).
33. Priestley, P. *et al.* Pan-cancer whole-genome analyses of metastatic solid tumours. *Nature* (2019) doi:10.1038/s41586-019-1689-y.
34. Drews, R. M. *et al.* A pan-cancer compendium of chromosomal instability. *Nature* **606**, 976–983 (2022).
35. Steele, C. D. *et al.* Signatures of copy number alterations in human cancer. *Nature* **606**, 984–991 (2022).
36. Duijf, P. H. G., Schultz, N. & Benezra, R. Cancer cells preferentially lose small chromosomes. *Int. J. Cancer* **132**, 2316–2326 (2013).
37. Gerstung, M. *et al.* The evolutionary history of 2,658 cancers. *Nature* **578**, 122–128 (2020).
38. Mendrzyk, F. *et al.* Isochromosome breakpoints on 17p in medulloblastoma are flanked by different classes of DNA sequence repeats. *Genes Chromosomes Cancer* **45**, 401–410 (2006).
